# Exploring ex vivo biofilm dynamics: consequences of low ampicillin concentrations on the human oral microbiome

**DOI:** 10.1038/s41522-024-00507-7

**Published:** 2024-04-02

**Authors:** N. K. Brar, A. Dhariwal, H. A. Åmdal, R. Junges, G. Salvadori, J. L. Baker, A. Edlund, F. C. Petersen

**Affiliations:** 1 Institute of Oral Biology, Faculty of Dentistry, University of Oslo, Oslo, Norway; 2 Department of Oral Rehabilitation & Biosciences, Oregon Health & Science University, Portland, OR USA; 3 Microbial and Environmental Genomics, J. Craig Venter Institute, La Jolla, CA USA; 4 Department of Pediatrics, UC San Diego School of Medicine, La Jolla, CA USA

**Keywords:** Microbial communities, Microbiology

## Abstract

Prolonged exposure to antibiotics at low concentration can promote processes associated with bacterial biofilm formation, virulence and antibiotic resistance. This can be of high relevance in microbial communities like the oral microbiome, where commensals and pathogens share a common habitat and where the total abundance of antibiotic resistance genes surpasses the abundance in the gut. Here, we used an ex vivo model of human oral biofilms to investigate the impact of ampicillin on biofilm viability. The ecological impact on the microbiome and resistome was investigated using shotgun metagenomics. The results showed that low concentrations promoted significant shifts in microbial taxonomic profile and could enhance biofilm viability by up to 1 to 2-log. For the resistome, low concentrations had no significant impact on antibiotic resistance gene (ARG) diversity, while ARG abundance decreased by up to 84%. A positive correlation was observed between reduced microbial diversity and reduced ARG abundance. The WHO priority pathogens *Streptococcus pneumoniae* and *Staphylococcus aureus* were identified in some of the samples, but their abundance was not significantly altered by ampicillin. Most of the antibiotic resistance genes that increased in abundance in the ampicillin group were associated with streptococci, including *Streptococcus mitis*, a well-known potential donor of ARGs to *S. pneumoniae*. Overall, the results highlight the potential of using the model to further our understanding of ecological and evolutionary forces driving antimicrobial resistance in oral microbiomes.

## Introduction

Since their widespread availability and use beginning in the late 1940s, antibiotics have saved millions of lives. However, the current rise of antibiotic-resistant infections represents a serious and growing threat to global health^1–3^. Multiple factors, including poor sanitation, limited access to clean water and a rise in global travel and migration, further contribute to transmission of drug-resistant microorganisms among populations^4–6^. Additionally, overuse and misuse of antibiotics creates a selective pressure where antibiotic susceptible bacteria are killed or inhibited, while antibiotic resistant bacteria survive^7–9^.

Historically, antibiotic resistance studies have focused on specific pathogens and antibiotic concentrations used to eliminate bacteria at infection sites. An issue that recently has gained attention is the collateral impact of antibiotics on the human microbiome at different anatomical sites. The biological response to an antibiotic drug depends on various pharmacokinetic factors^7,10^. When antibiotics reach the distinct sites within the human body, the concentrations of the drug differ in time and space, often resulting in prolonged exposure to low antibiotic concentrations. In addition, the impact of antibiotics on the microbiome may be affected by how microbes are organized, with microbial composition and biofilm mode of growth playing an important role. Microbes organized in biofilms are generally less susceptible to the effect of antibiotics. There are two primary concerns related to the off-target effects of antibiotics on biofilm communities. Firstly, there is the issue of the loss of colonization resistance, which refers to the mechanism by which the commensal microbiome’s regular activities prevent growth of pathogens^11^. Secondly, there is the major concern of the enrichment for antimicrobial resistance genes (ARGs) and drug-resistant bacteria in the human microbiome^12,13^.

Most of the research reporting on the impact of antibiotics on the human microbiome and the associated ARGs (resistome) has focused on the gut. Results of these studies vary with some reporting an increase in ARG load, while others demonstrate no effect. Results for changes in ARG diversity also differs, varying from slightly increased diversity to no effect, and some studies showing decreased richness^14–16^. Multiple factors have been proposed to explain these differences, such as variations in the populations studied, the type of antibiotics used, age differences, and a range of other factors^16–19^. Although less well-studied in this context, the oral microbiome is thought to be less prone to changes in microbial ecology due to antibiotic treatment than the gut^20,21^. The oral cavity and adjacent anatomical regions are, however, important ecological niches that serve as reservoirs for the emergence and dissemination of antibiotic resistance genes and antibiotic-resistant bacteria, often thriving in the form of biofilms^22–25^. Four of the 12 WHO Global priority pathogens are often found in the oral cavity including; *Streptococcus pneumoniae*, *Haemophilus influenzae*, *Klebsiella pneumoniae*, and *Staphylococcus aureus*^26^.

One of the most worldwide prescribed antibiotics by clinicians and other healthcare professionals is ampicillin^27,28^. This is a broad-spectrum antibiotic that interferes with the cross-linkage of peptidoglycans in the bacterial cell wall to inactivate and kill bacteria^29^. Ampicillin is widely used to treat infections in the respiratory tract and other body sites^30^. When administered orally, ampicillin concentration in saliva can reach peak concentrations close to or higher than minimum inhibitory concentrations (MIC) for several of the most studied bacteria in the oral cavity^31,32^. At concentrations below the MIC, ampicillin and several other antibiotics have shown to stimulate biofilm formation by *S. aureus* and *H. influenzae*, as well as by other pathogens found in the oral cavity and at close anatomical sites, such as *Streptococcus intermedius* and *Enterococcus faecalis*^13,33–36^.

To address the inherent limitations of human studies related to multiple confounding factors, various ex vivo models have been proposed^37–40^. Such models are particularly relevant to generate hypotheses for further in vivo investigations. So far, they have been applied mostly to better understand ecological changes associated with specific disease states^40–42^. Here, we investigated the ecological impact of ampicillin on the oral microbiome using an ex vivo highly reproducible biofilm model of the human oral microbiome^43^. Our focus was on the effect of ampicillin on viability and microbiome composition, alongside assessing changes in diversity, composition, and abundance of ARGs. Our results showed an increase in biofilm viability by low ampicillin concentrations and an unexpected decrease in ARG abundance by both low and high concentrations.

## Results

### The impact of different ampicillin concentrations on oral biofilm viability

**Fig. 1:**
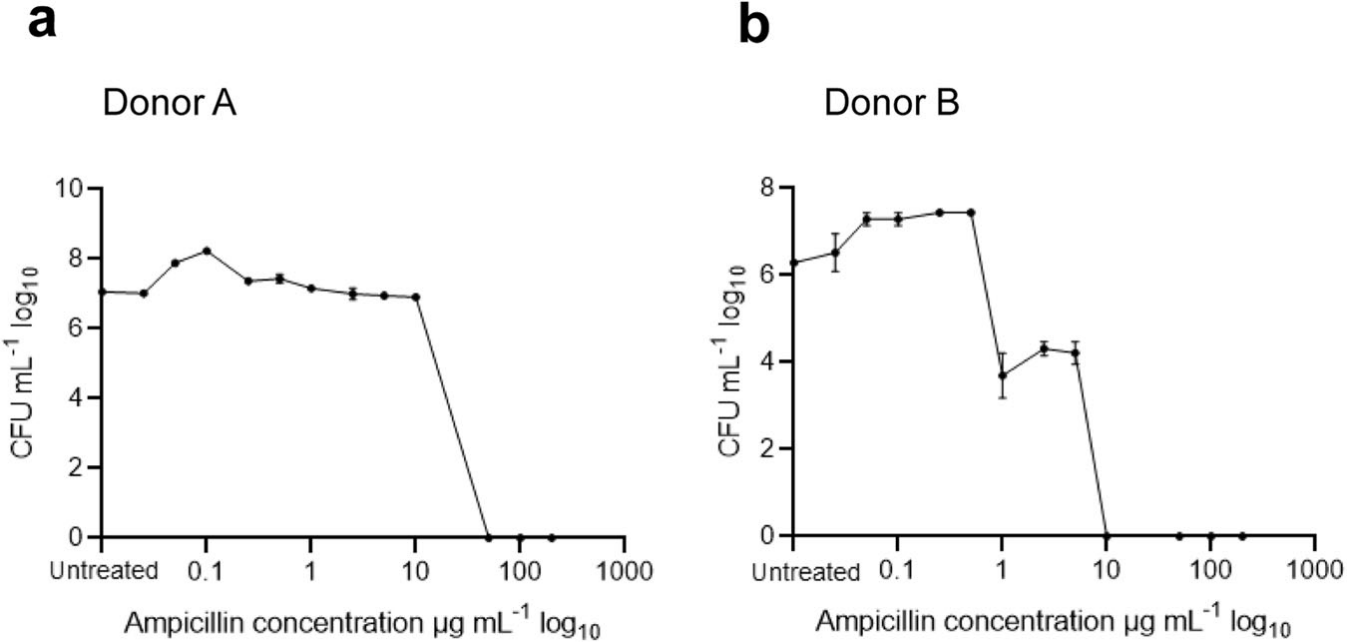
Oral microbiome viability following treatment with different ampicillin concentrations. Numbers of viable cells in the community, as determined by colony-forming units counted on SHI agar plates for (**a**) donor A and (**b**) donor B. The data are shown for triplicate experiments as mean ± SE. The untreated samples was given a value of 0.01 to adjust it to the log-scale for visualization purposes. Biofilms were grown for 48 h.

Ampicillin ranging from 0 to 200 µg ml^−1^ was used to investigate the impact on biofilm viability using saliva inoculum from two different donors (Fig. 1). Despite some variation in the curve response shape for each of the donor samples, low concentrations, including 0.05 µg ml^−1^ and 0.1 µg ml^−1^, resulted in increased biofilm viability by ~1.2 log_10_ compared to the negative control. The viability decreased in biofilm samples at high concentrations until viable cells could no longer be detected.

### The impact of low ampicillin concentrations on oral microbiome ecology

**Fig. 2:**
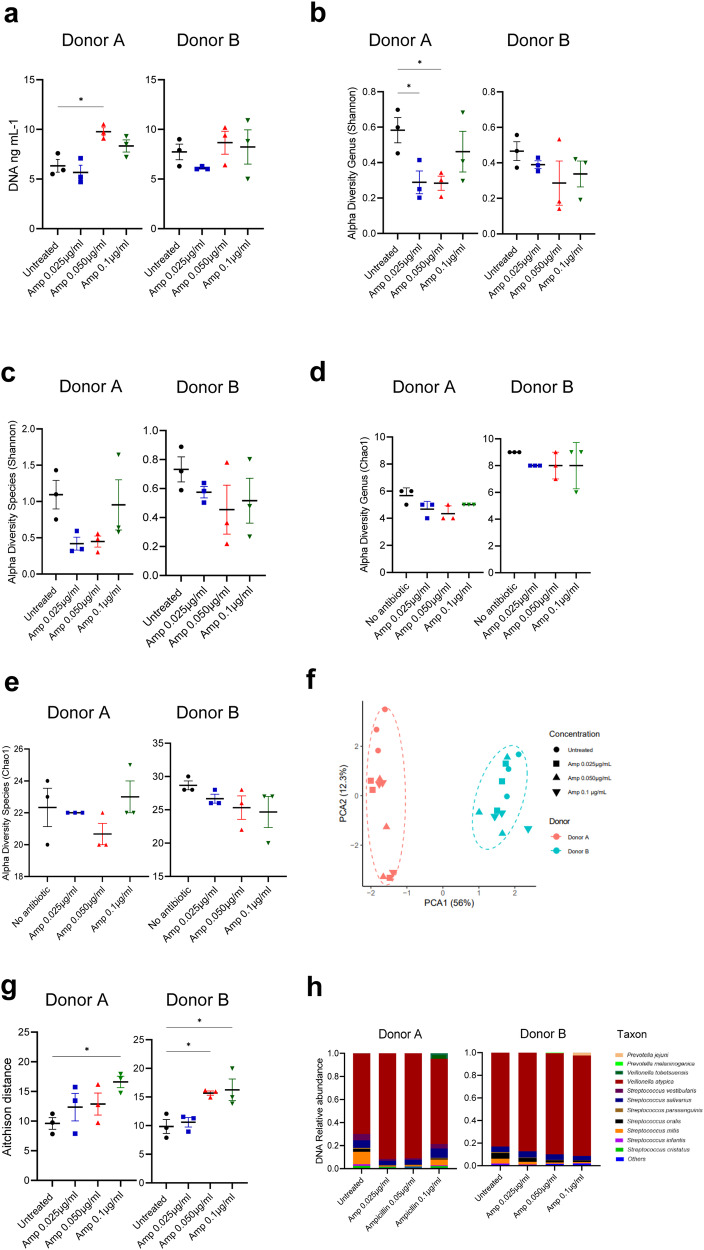
The effect of low ampicillin concentrations on the microbiome of ex vivo oral biofilm communities. **a** DNA concentration measured by Qubit4 in ng ml^−1^. Alpha-diversity measured by Shannon index which indicates richness and evenness on (**b**) genus and (**c**) species level. Alpha diversity measured by Chao1 indicates the total richness at (**d**) genus level and (**e**) species level. **f** Principal component analysis (PCA) ordination plot with Aitchison distance illustrating beta-diversity. **g** Dissimilarity in oral microbiome within each treatment group, each point shown as Aitchison distance. **a**–**e**, **g** Error bars represent mean SEM. One-way ANOVA followed by Benjamini–Hochberg (BH) post hoc test, *p < 0.05. **h** Stacked bar plots display the relative abundance of the 12 most abundant species in the microbiomes.

In total, 24 samples from donors A and B were analyzed by shotgun metagenomic sequencing to investigate the impact of 0.025, 0.05 and 0.1 µg ml^−1^ ampicillin on oral microbiome ecology. These concentrations were chosen based on clinical data reporting levels up to 0.12–0.28 µg ml^−1^ ampicillin in saliva followed by oral administration^31,32,44^. They were also within the range in which biofilm viability was enhanced (Fig. 1). Compared to the untreated samples, there was a significant increase in DNA concentration for donor A in response to antibiotic treatment. Although not statistically significant, a similar trend was observed for donor B (Fig. 2a).

A total of 234 million (M) paired reads across all samples were obtained after quality filtering, with an average of 9.8 M-reads per sample (minimum of 3.9 M and maximum of 19.9 M reads per sample). In total, 48 bacterial species were detected across all samples.

Alpha diversity (intra-sample diversity) using the Shannon index, which takes into account both taxonomic abundance and evenness, revealed a significant reduction in alpha diversity at genus level for donor A, but not donor B, compared to the untreated samples (Fig. 2b). At species level, a tendency in alpha-diversity reduction, although not statistically significant, was also observed for both donors (Fig. 2c). No significant differences were observed for either donor A and B using Chao1 index for alpha diversity at genus and species level, which takes into account only microbial taxonomic richness (Fig. 2d, e). Beta diversity (inter sample diversity) showed a significant difference between the two donors (*p* = 0.001, *R*^2^ = 0.548, *F* = 26.706, permutational multivariate analysis of variance [PERMANOVA]) (Fig. 2f). Compared to the control, a significant increase in microbiome dissimilarity was observed for the highest ampicillin concentrations for both donors (Fig. 2g).

**Fig. 3:**
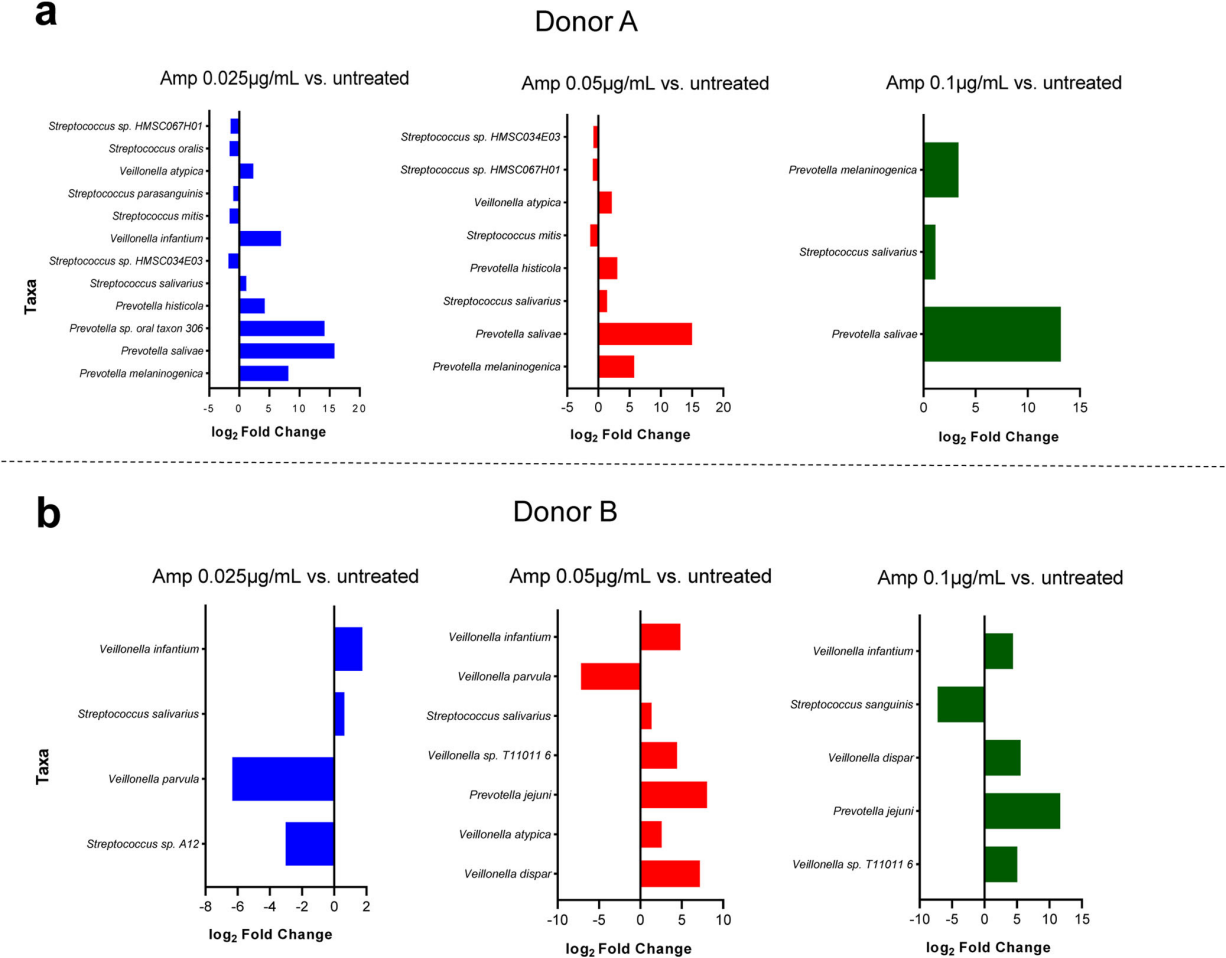
Species with significantly different abundances upon treatment with low concentration of ampicillin. Bar charts illustrate the log_2_ fold change of taxa, adjusted for false discovery rate (FDR), *p* values < 0.05. **a** Donor A and (**b**) donor B (based on DESeq2).

The relative taxonomic composition varied with respect to ampicillin treatment compared to the non-treated controls. The untreated samples in both Donor A and B showed *Veillonella atypica* being the most abundant species (Fig. 2h and Supplementary Table 1). Differential abundance analysis using DESeq2 revealed a statistically significant increase in gram-negative bacteria within the genus *Veillonella* and *Prevotella* for the antibiotic-treated biofilm samples from both donors (Fig. 3). For donor A, *V. atypica*, *V. infantium*, *P. jejuni*, *P. histicola*, *P*. sp. Oral taxon 306*, P. salivae* and *P. melaninogenica* increased significantly. In contrast, *Streptococci* such as *S. oralis*, *S. mitis, S. parasanguinis, S*. sp. HMSC034E03*, S* sp. HMSC067H01 were reduced. In donor B, only *V. atypica*, *V. infantium, V. dispar, V*. sp T11011 6 and *P. jejuni* increased significantly. On the other hand, *V. parvula*, *S*. sp.12 and *S. sanguinis* were decreased. The only *Streptococcus* species which showed a significant increase under ampicillin treatment in both donors was *S. salivarius*. Among the pathogen priority list by the WHO, we found *S. pneumoniae* in all samples from donor A, and *S. aureus* in donor B. Their relative abundance was not significantly changed in ampicillin treated samples at all concentrations (Supplementary Table 1).

### The impact of low ampicillin concentrations on oral resistome

A total of 129,258 paired reads were annotated as ARGs across all samples, with an average of 5385 reads (min–max:1620–13,067) per sample. In total, 28 ARGs were detected. The ARGs were found to belong to 10 different antibiotic drug classes associated with three different mechanisms of resistance; antibiotic efflux, antibiotic inactivation and antibiotic target protection (Supplementary Table 2).

**Fig. 4:**
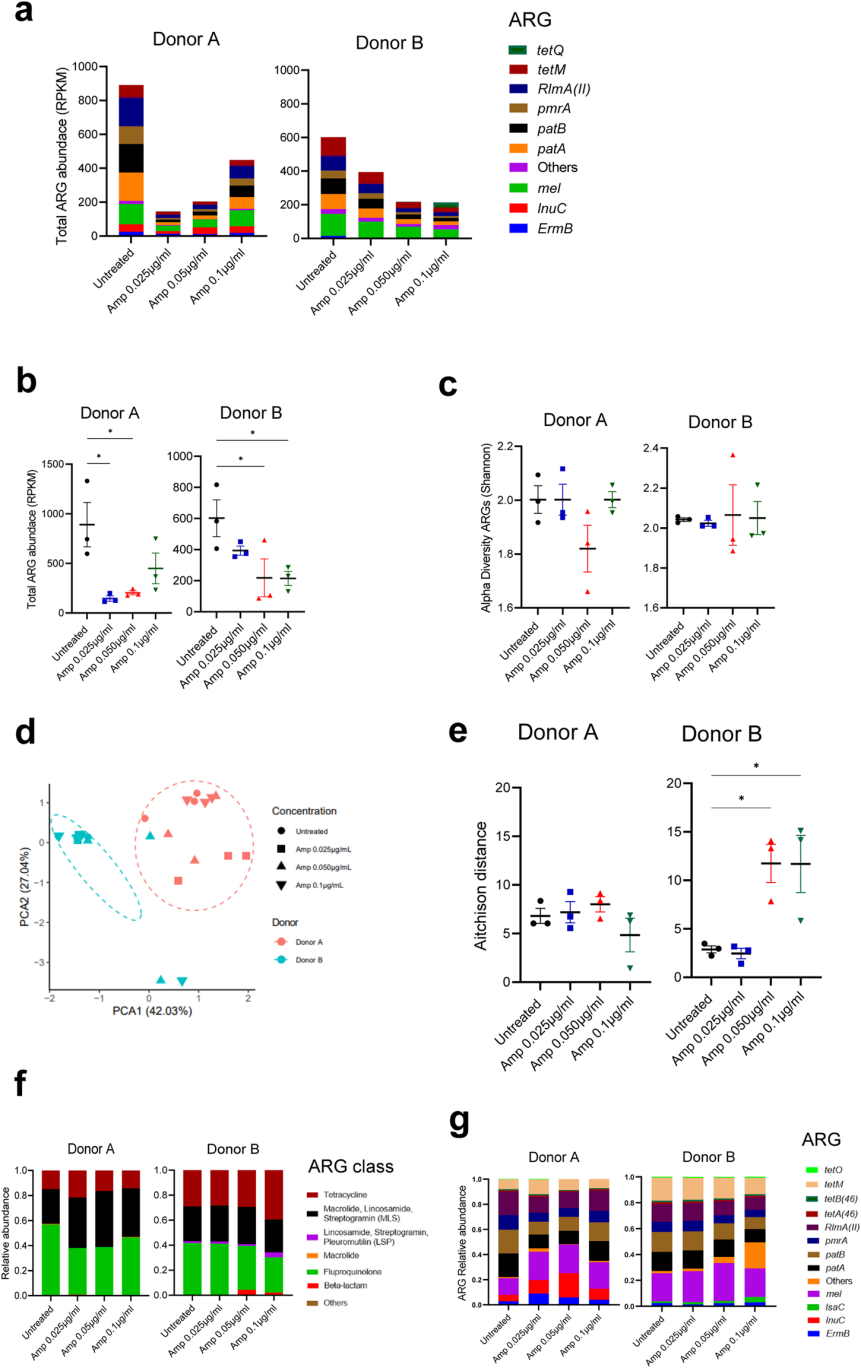
The effect of low ampicillin concentrations on the resistome of ex vivo oral biofilm communities. **a** The total ARG abundance visualized as reads per kilobase million values (RPKM) in the oral biofilm community. **b** Total mean RPKM display error bars with mean SEM. One-way ANOVA followed by Benjamini–Hochberg (BH) post hoc test. *p < 0.05. **c** Alpha diversity measured at ARG level by Shannon index which indicates richness and evenness. **d** Principal component analysis (PCA) ordination plot with Aitchison distance illustrating beta-diversity. **e** Dissimilarity in oral resistome within each treatment group, each point shown as Aitchison distance. Error bars represent mean SEM. One-way ANOVA followed by BH post hoc test. *p < 0.05. Stacked bar plots displays the relative abundance of (**f**) all antimicrobial classes and (**g**) the 12 most abundant antimicrobial resistance genes (ARGs) found in the oral biofilm community.

For both donors, treatment with low concentration of ampicillin decreased ARG abundance compared to the untreated samples, visualized by bar plots for each ARG (Fig. 4a). The reduction in ARG load by ampicillin was statistically significant for both donors (Fig. 4b). Compared to the untreated samples, both donor A and B failed to show major changes in ARG alpha diversity (Fig. 4c). For beta diversity, the samples clustered according to individuals (*p* = 0.001, *R*^2^ = 0.3357, *F* = 11.117), permutational multivariate analysis of variance [PERMANOVA]) (Fig. 4d). A significant increase in oral resistome dissimilarity was observed within the samples exposed to ampicillin compared to the control in donor B, but not donor A (Fig. 4e).

The most abundant ARG classes detected in both donors were fluoroquinolone, as well as macrolide, lincosamide, streptogramin (MLS) followed by tetracycline in both control and ampicillin treated samples (Fig. 4f). Donor A showed an increased abundance of the *mel* gene in ampicillin treated samples, which is associated with macrolide resistance. In addition, some beta-lactam genes such as *PC1* and *CfxA3* were detected only in samples treated with ampicillin in donor A. Oral biofilms treated with ampicillin in donor B harbored more beta-lactam genes in *CfxA* family, compared to the untreated control (Fig. 4g and Supplementary Table 3).

### Association between the oral microbiome and resistome

**Fig. 5:**
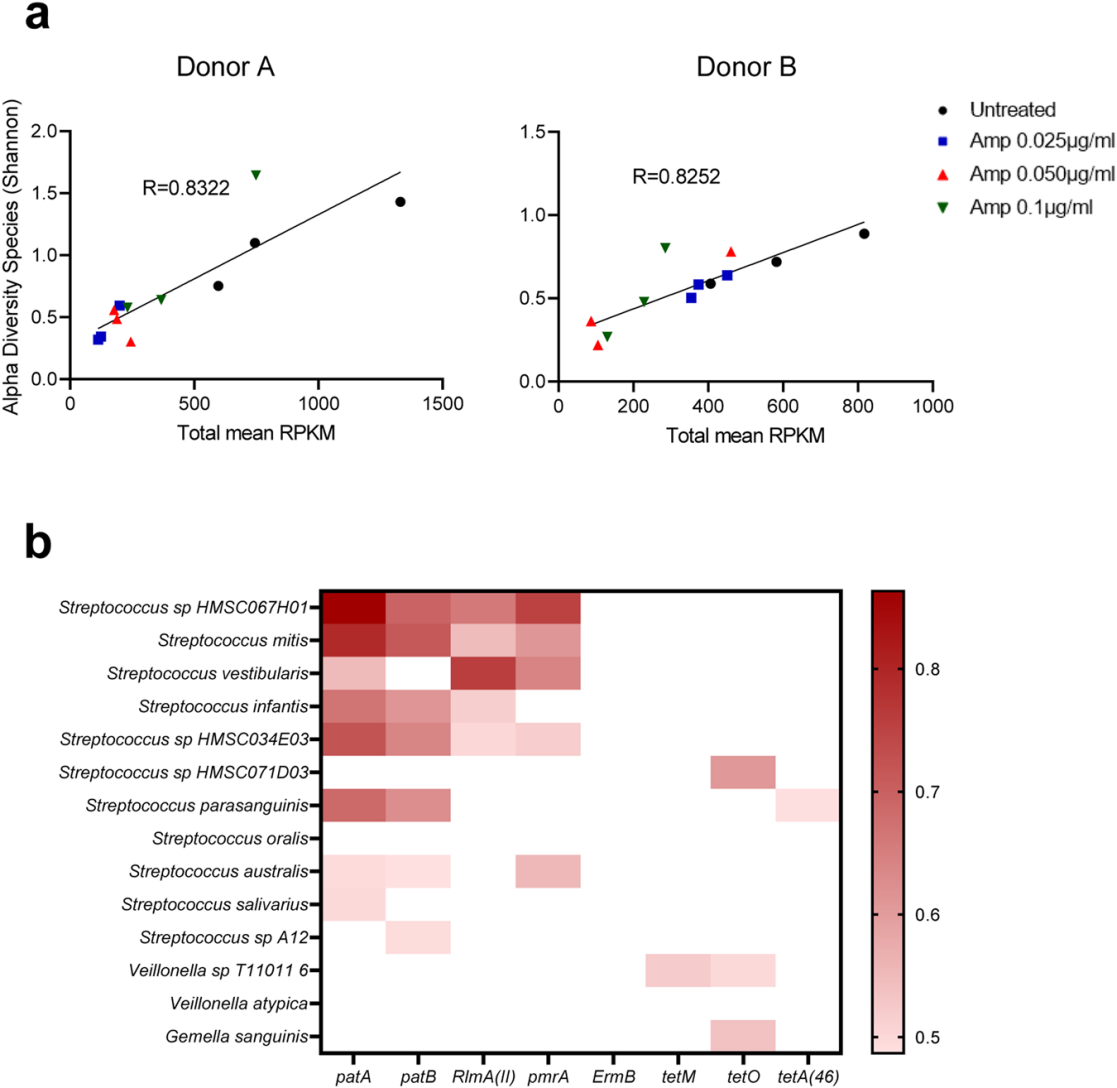
Correlations between the oral microbiome and resistome. **a** Spearman correlation matrix between microbiome alpha-diversity and ARG in RPKM. Correlation coefficients “R” were between 0.8–1 showing positive correlation between the two variables (*p* < 0.001). **b** Heatmap represents pairwise Spearman correlation between ARG and bacterial species abundance from oral biofilms in Donor A and B. Correlation cut off was 0.1. Benjamini–Hochberg method was used to adjust for multiple comparisons. Adjusted for false discovery rate (FDR), *p* values < 0.05.

A Spearman correlation matrix revealed a strong positive correlation between ARG abundance and microbial alpha-diversity (Shannon index, species level) in donor A (*R* = 0.8322) and donor B (*R* = 0.8252) (Fig. 5a). To predict the origin of ARGs, Spearman’s pairwise correlation analysis was also conducted between ARGs and species abundance (Fig. 5b). Efflux pump ARGs such as *patA*, *patB* and *pmrA* were strongly correlated with *Streptococcus* species, including *S*. sp. HMSC067H01, *S. mitis*, *S. vestibularis*, *S. infantis*, *S*. sp. HMSC034E03, *S. parasanguinis*, *S. australis* and *S. salivarius*. A positive correlation between increased *Veillonella* sp. and tetracycline resistant genes *tet(O)* and *tet(M)* was also observed indicating *Veillonella* sp. as the potential host for *tet(O)* and *tet(M)*.

### The impact of ampicillin on the microbiome and resistome by high ampicillin concentration

Although the primary focus of the study was on low ampicillin concentrations, an additional experiment to investigate the impact of a high concentration was performed. Saliva samples from a third donor (donor C) were first investigated for the impact of ampicillin at different concentrations on biofilm viability. This was chosen as no more samples from donor A and B were available. Here, similar to donors A and B, we also found that low concentrations favored biofilm viability (Supplementary Fig. S1). Results for a high concentration (~10 μg ml^−1^), showed a significant reduction in DNA concentration compared to the control (Supplementary Fig. S2). No significant difference was observed in alpha diversity (Supplementary Fig. S2). Compared to the untreated samples *V. atypica* reduced significantly. In contrast, there was an increase in the relative abundance of *Streptococcus* species, including *S. salivarius*, *S. parasanguinis* and *S. infantis* (Supplementary Fig. S2). Among the pathogen priority list by the WHO, we found *S. pneumoniae* in all samples from donor C (Supplementary Table 4).

Results from oral resistome revealed a tendency for a decrease in ARG load and an increase in ARG alpha diversity in the treated samples compared to the control, although these findings were not statistically significant (Supplementary Fig. S3a, b and Supplementary Table 5). A relative increase was observed for ARGs such as *mel*, *tetA*(46), *tetB(*46) and others, while *patA*, *patB* and *pmrA* reduced (Supplementary Fig. 3c, d and Supplementary Table 6).

## Discussion

Sub-inhibitory concentration of antibiotics can impact bacterial gene expression, and trigger behaviors involved in virulence, such as biofilm formation, quorum sensing, and horizontal gene transfer (HGT)^13,34,45,46^. Most research on sub-inhibitory concentrations of antibiotics has been conducted on single bacteria and rarely on a consortium of defined microbial species^47,48^. Here we demonstrate that ampicillin, at low concentrations, favored biofilm viability within a diverse oral microbial community. An increase in bacterial DNA was also observed, which may represent both viable, non-viable, or extracellular DNA in the biofilms^49,50^. To the best of our knowledge, this is the first study to report antibiotic effects in an ex vivo human microbiome model combining biofilms and shotgun metagenomics. The metagenomic changes induced by ampicillin were highly reproducible between replicates from the same donor and showed donor-specific clustering features in line with inter-individual variations characteristic of human microbiomes^51,52^.

The oral microbiome is best understood within the framework of the most prevalent oral diseases, namely dental caries and periodontal disease. However, the oral microbiome also represents an important reservoir of pathogens and antibiotic resistance genes^53–56^. *S. pneumoniae*, *K. pneumoniae*, *H. influenzae* and *S. aureus*, for instance, are mostly often thought of as residents of the nasopharynx, but their presence in the oral cavity is of relevance, particularly due to saliva being a main route for dissemination of microorganisms between humans, both via direct contact and droplets. In saliva samples collected from healthy donors for this study, *S. pneumoniae* was identified in the samples from two of the three donors. Previous studies have also demonstrated *S. pneumoniae* in biofilms using a similar ex vivo model as the one used in our study^43,48^. We found that none of the concentrations used resulted in changes in *S. pneumoniae* abundance. Yet, among the 12 most prevalent antibiotic resistance genes, four were correlated with *S. mitis* and other oral streptococci closely related to *S. pneumoniae*. Of note, clinical and laboratory data indicate that oral streptococci are an important reservoir of antibiotic resistance genes that can be readily transmitted to *S. pneumoniae* by horizontal gene transfer via natural transformation, thus comprising treatment of invasive pneumococcal diseases^57–59^. In line with in vivo studies, oral streptococci were also among the most prevalent bacteria in our model, independent of donor, indicating the possibility of using variations of the current model to investigate this important phenomenon in complex communities that approximate in vivo conditions. Another WHO priority pathogen, *S. aureus*, was also found in all samples from one of the donors exposed to low ampicillin concentration, but its abundance remained unchanged.

The variations in microbiome and resistome composition among the donors in our model align with the well-known complexity and variability of the human microbiota^60–62^. Despite such individual differences, we discovered a decrease in ARG load that was common to all donor samples, and also with ampicillin concentrations that had no inhibitory (donors A and B) or inhibitory (donor C) effects on overall bacterial viability. For the low concentrations, the dynamics of inhibition varied, with donor A samples displaying a decrease that was directly related to the ampicillin concentration employed, whereas donor B exhibited a reduction that showed an inverse correlation. This underscores the intricate and individual nature of microbiomes.

The reduction in antimicrobial resistance gene load by both low and high ampicillin concentration was somewhat surprising. However, most available studies using metagenomics have focused on the gut resistome. In these studies, the reported outcomes on the resistome vary from no significant effects to increased antibiotic resistance gene load and diversity following antibiotic therapy^21,63–65^. For the oral microbiome, our knowledge is mostly restricted to a few studies indicating that the oral microbiome is more resilient to changes than the gut microbiome^20,21^. Reports on the resistome are mostly based on functional predictions using 16S rRNA gene data^21^. Resilience of the oral microbiome can be a result of evolutionary processes by having evolved in the presence of mechanical disrupting forces from salivary flow and mastication, as well as fluctuations in diet, temperature, and chemical agents^66^, or that oral biofilms may be more impermeable to antimicrobials. Alternatively, pharmacodynamics and pharmacokinetics of antibiotics can also be relevant as for at least some antibiotics, the concentrations that reach saliva following absorption are lower than in the gastro-intestinal tract^16,60^. Also, in our model the ecological effects were studied after 24 h of antibiotic exposure, while in clinical studies on the oral microbiome and resistome the reported effects are after 7 days antibiotic course^60–62^. Investigating the possibility that the observed reduction in antibiotic resistance gene load in our study may occur also in vivo during shorter-courses warrants further investigations, since such a finding would provide relevant information for antibiotic-stewardship programs aiming at reducing the length of antibiotic therapies^67^. Additionally, it is important to acknowledge that the specific antibiotic used in our study was ampicillin, implying that different antibiotic choices may yield different results.

Another interesting finding was the increased dissimilarity in microbiome composition within the samples exposed to low antibiotic concentrations compared to the control. Such findings have also been observed in human clinical studies^63^. Increased dissimilarity in resistome composition was also observed, but only for one of the donors. This is an interesting phenomenon that is not yet understood or universally proven, but that may relate to stochastic mechanisms involved in the response to antibiotics^9^.

Overall, the results indicate that the model can be useful for future studies investigating the impact of different antibiotics or examining other interventions with the potential to alter the ecology and evolution of antimicrobial resistance in oral biofilms, and that include main pathogens listed by the WHO as priority microorganisms for controlling antimicrobial resistance^26^. The current model has previously been adapted to investigate conditions such as caries by changing the carbohydrate substrate used in the growth medium^41^, or from patients with periodontal disease to obtain a specific disease-state sub gingival community^42^. For *S. pneumoniae*, for instance, samples could be from children, as young age is associated with prevalent carriage of pneumococci, and these are one of the most vulnerable age groups to pneumococcal infections^68^. Of notice, recent studies using prediction models estimate that exposure of *S. pneumoniae* and other pathogens found in the oral cavity and adjacent anatomic regions to antibiotics are in more than 90% of the cases when they are not the target of the antibiotic therapy^7^. This highlights the importance of advancing our understanding of the impact of antibiotics on antimicrobial resistance from an ecological and evolutionary perspective. Ex vivo models are particularly relevant as they are useful for mechanistic studies and are not subjected to main ethical issues. Since the environment is stable and controlled, it has the potential to generate highly reproducible results, thus avoiding the shortcomings of high variability observed in clinical studies of human microbiomes^43^. In addition, since shotgun approaches are still costly and will require large clinical data before finding its way from bench to bed side, modeling can be a cost-effective way to help develop new hypothesis and predictive models to test against available and future human data sets.

## Methods

### Sample collection

The study was conducted in accordance with the Declaration of Helsinki and approved by the National Regional Ethics Committee (REK20152491) for studies involving human samples. A written informed consent was obtained from the donors to participate in the study. The participants were asked to brush their teeth after breakfast and refrain from any food or drink 2 h before donating saliva. They rinsed the mouth three times with water 10 min prior to saliva collection. Non-stimulated saliva was collected from each person. Saliva samples were centrifuged at 6000 × *g* for 5 min at 4 °C to spin down large debris and eukaryotic cells. The supernatant was used as saliva derived inoculum. Cell-free saliva was obtained by centrifuging saliva samples at 10,000 × *g* for 7 min at 4 °C. The upper fraction was used as pellicle to coat the bottom of the wells prior to growing the biofilms as previously described^43^. Saliva from three donors was used in the study. All experiments were conducted in triplicates, including controls and ampicillin treated biofilms.

### The human oral microbiome biofilm model

A previously described ex vivo biofilm model that maintains a highly reproducible species and metabolic diversity of the human oral microbiome was utilized^43,69^. Briefly, SHI media was pre-reduced for 4 h in anaerobic conditions (carbon dioxide, 5%; balanced with nitrogen)^70^. Saliva samples were used to inoculate the SHI media (2 µl saliva/ ml), which was then distributed to the wells of a 24-well plate (1 ml per well) pre-coated with a saliva pellicle, followed by incubation in an anaerobic chamber at 37 °C for 24 h. The liquid phase was then removed and fresh SHI medium was added to the pre-formed oral biofilms. Samples were either not treated (control) or treated with ampicillin ranging from 0.025–200 µg ml^−1^ (Sigma-Aldrich). The ampicillin stock (50 mg ml^−1^ in distilled water) was diluted in SHI medium before adding to the biofilms. After 48 h, the oral biofilms were washed and resuspended in 1 ml PBS, and 20% glycerol was added before the samples were stored at −80 °C.

### Oral biofilm viability assay

For biofilm viability assessment, the samples obtained as described above from controls and treated biofilms were diluted in 10-fold dilution series, and 20 µl of each dilution was then plated on SHI agar plates. The plates were incubated for 48 h at 37 °C in the anaerobic chamber to calculate colony-forming units per milliliter (CFUs ml^−1^ log_10_).

### DNA extraction

Bacterial DNA was extracted using the MasterPure^TM^ Gram Positive DNA Purification Kit (Epicentre, Madison, WI, USA) using the manufacturer’s protocol. Precipitated DNA was resuspended in 35 µl milliQwater. A NanoDrop^TM^ 2000c spectrophotometer (Thermo Fisher Scientific, Waltham, MA, USA) and a Qubit^TM^ 4 Fluorometer (Thermo Fisher Scientific, Waltham, MA, USA) were used to measure the quality and the amount of extracted DNA.

### DNA library preparation and sequencing

DNA library preparation was conducted using an Illumina DNA Prep kit, (m) (Illumina, Inc., San Diego, CA, USA) following the manufacturer’s protocol. The final DNA library was retrieved by resuspending it in the provided buffer, and each sample adjusted to 500 ng in 30 µl using nuclease-free water. The quality and the concentration of the DNA library was measured using a NanoDrop^TM^ 2000c spectrophotometer and Qubit ^TM^ 4 Fluorometer, and further analyzed using a Bioanalyzer (Agilent Technologies, Santa Clara, CA, USA) and a High Sensitivity DNA kit (Agilent Technologies, Santa Clara, CA, USA) using the manufacturer’s protocol. Metagenomic shotgun sequencing was conducted at the Norwegian Sequencing Centre (Oslo, Norway) using Illumina 3000/4000 High Seq (Illumina, Inc., San Diego, CA, USA).

### Quality control and pre-processing of metagenomic data

The quality of raw and preprocessed sequencing reads was evaluated by FastQC tool (v.0.11.8)^71^. Low quality reads and adapter sequences were trimmed using Trimmomatic (v.0.35)^72^ with the following parameters: ILLUMINACLIP: Nextera PE:2:30:10 LEADING:3 TRAILING:3 SLIDING WINDOW:4:15 MINLEN:36. Reads which mapped to the human genome (GRch38) using Bowtie 2 (v.2.3.4)^73^ were removed. The remaining high-quality reads were then subjected to microbiome and resistome profiling.

### Taxonomic and resistome profiling

MetaPhlAn (v.2.0)^74^ was used to profile the bacterial composition in the oral biofilm samples and to determine their abundance at the species-level. For ARG prediction, reads were mapped against the Comprehensive Antibiotic Resistance Database (CARD) using the Bowtie 2 alignment tool^75,76^. ARGs with >80% gene fraction (proportion of nucleotides that align with at least one read to the reference ARG) were considered to be positively detected in a sample. Read counts were normalized for differences in both gene lengths and bacterial abundances for each sample by calculating reads per kilobase of reference gene per million bacterial reads (RPKM).

### Downstream analysis

MicrobiomeAnalyst^77,78^ and ResistoXplorer^79^ were used to carry out comprehensive exploration, analysis and visualization of the microbiome and resistome count data. GraphPad (Prism 9 software) and R (v3.6.0) were used for graphical representation and statistical analysis. Alpha diversity using the Shannon and Chao1 diversity indexes were calculated at the genus, species and ARG level. Beta diversity was estimated using Aitchison distance on centered log-ratio (CLR) transformed counts on species level, using the *ordinate* function from the phyloseq (v.1.34.0) R package^80,81^, and visualized in a compositional principal component analysis (PCA) ordination plot. Differences in beta diversity were tested using permutational multivariate analysis of variance (PERMANOVA) using the *adonis* function (vegan (v.2.5.7) package) with 999 permutations. The dissimilarity in oral microbiome and resistome composition within replicates were calculated using the Aitchison distance. Pairwise comparisons of log-fold change in abundance between groups were performed using DESeq2 (v.1.36.0)^81^, adjusted for multiple comparisons using the Benjamini–Hochberg (BH) procedure. We conducted the association analysis based on the pairwise Spearman’s correlations among microbial taxa and ARG using the integration module in ResistoXplorer. One-way analysis of variance (ANOVA) adjusted for multiple comparisons using the Benjamini–Hochberg (BH) procedure and two-tailed unpaired *t*-test were used to compare group differences, as appropriate. *p* values of <0.05 were considered statistically significant.

### Reporting summary

Further information on research design is available in the Nature Research Reporting Summary linked to this article.

### Supplementary information

Supplementary material

Reporting summary

## Data Availability

The metagenomic dataset is available at the NCBI Sequence Read Archive under accession BioProject PRJNA773113.
