# Supplementary material for: Exploring ex vivo biofilm dynamics: consequences of low ampicillin concentrations on the human oral microbiome
Source: NPJ Biofilms Microbiomes. 2024 Apr 2;10:37. doi: 10.1038/s41522-024-00507-7 (PMC10987642; doi:10.1038/s41522-024-00507-7)
Supplement: Supplementary file 1 — Supplementary material [file 41522_2024_507_MOESM1_ESM.pdf]

Donor C

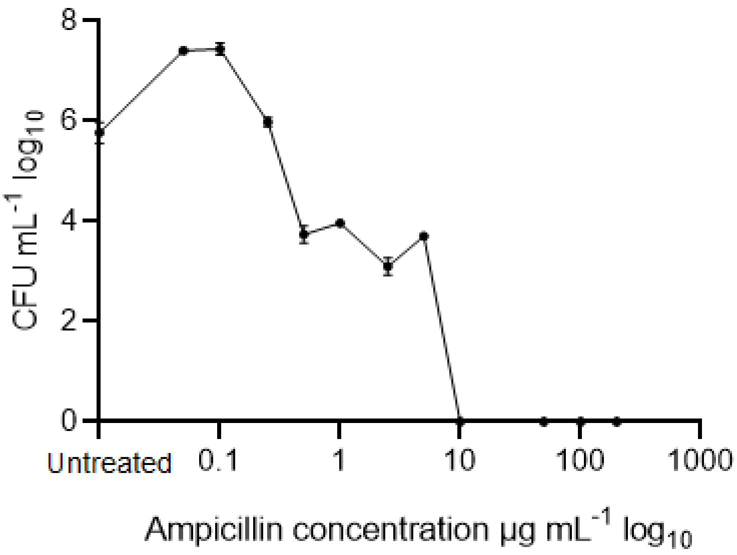

Supplementary Figure 1: Oral microbiome viability following treatment with different ampicillin concentrations. Numbers of viable cells in the community, as determined by colony-forming units counted on SHI agar plates for donor C. The data are for triplicate experiments. The data are shown for triplicate experiments as mean ± SE. The untreated samples was given a value of 0.01 to adjust the log-scale for visualization purposes.

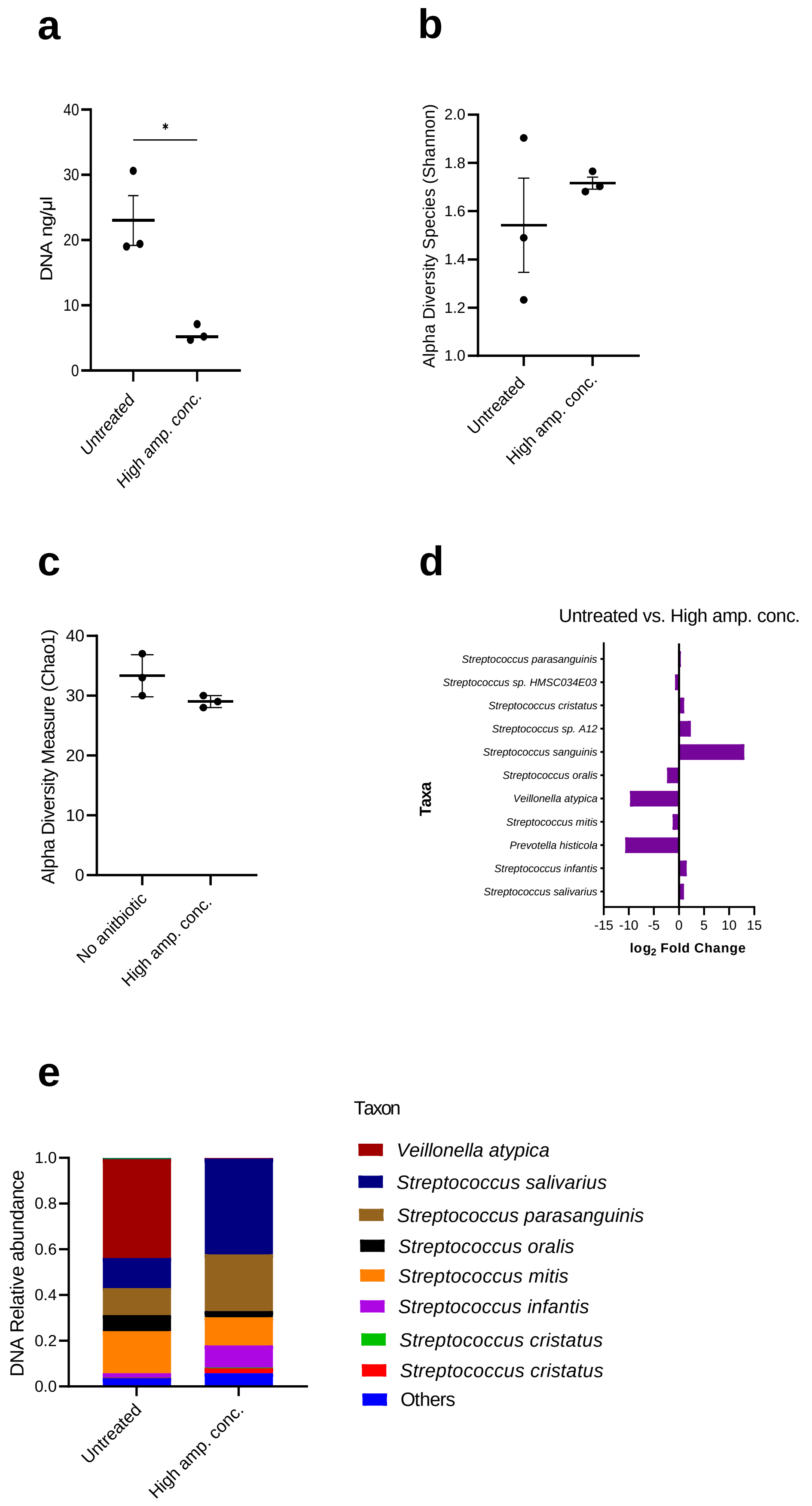

Supplementary Figure 2: The effect of high ampicillin concentration on the microbiome of *ex vivo* oral biofilm communities. (a) DNA concentration measured by Qubit4 in ng mL^{-1}. (b) Alpha-diversity measured by Shannon index indicates richness and evenness on species level. (c) Alpha diversity measured by Chao1 indicates the total richness at species level. (a-c) Two –tailed unpaired t-test, **P*<0.05. Error bars represent mean SEM. (d) Bar charts illustrates the $\log_2$ fold change of taxa adjusted for false discovery rate (FDR), *p*-values <0.05 (based on DESeq2). (e) Stacked bar plots display the relative abundance of the 12 most abundant species in the microbiomes.

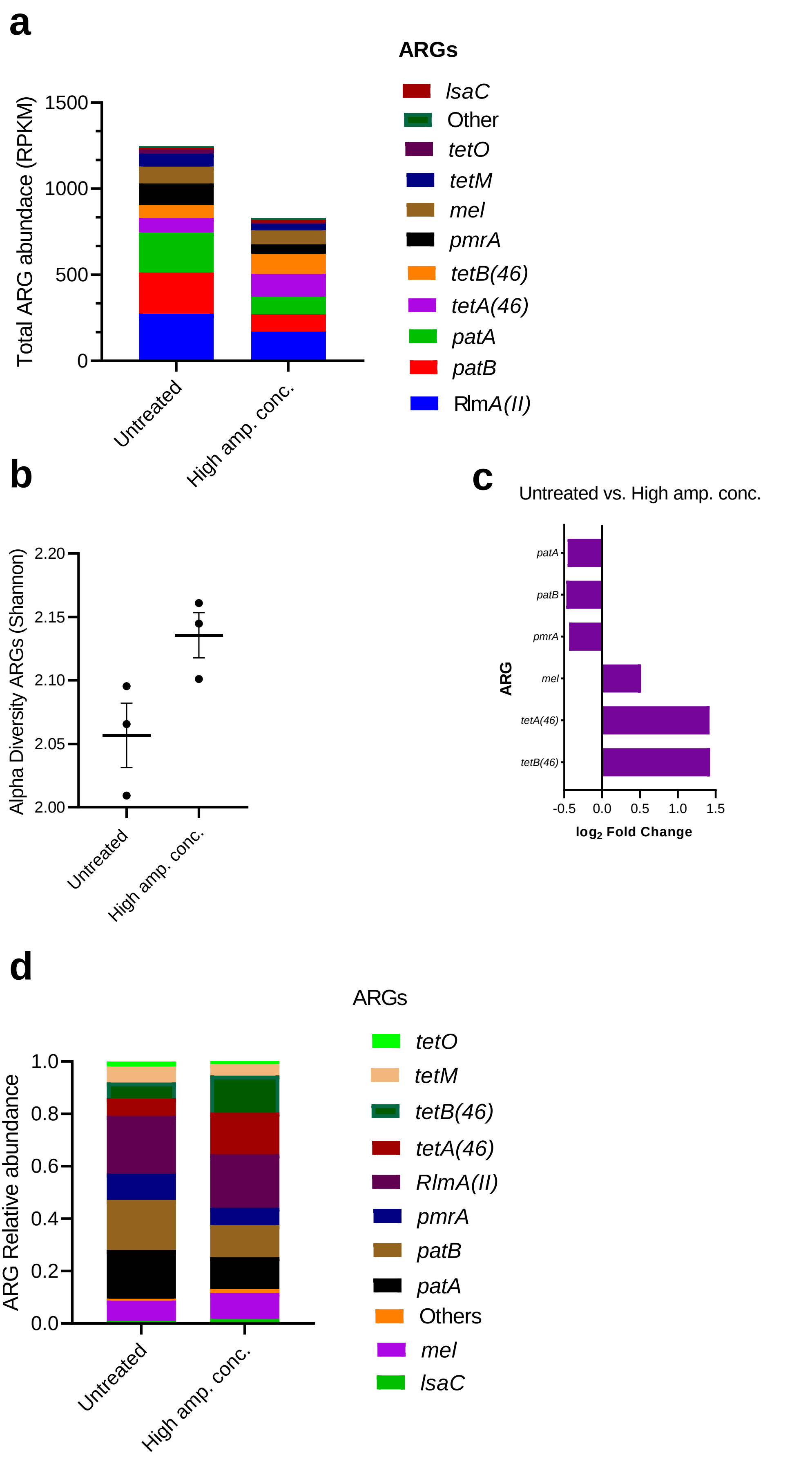

Supplementary Figure 3: The effect of high ampicillin concentration on the resistome of *ex vivo* oral biofilm communities. (a) ARG abundance visualized as reads per kilobase million values (RPKM) in the oral biofilm community. (b) Alpha diversity measured at gene level by Shannon index which indicates richness and evenness. (c) Stacked bar plots displays the relative abundance of the 12 most abundant antimicrobial resistance genes (ARGs) in the resistome. (d) Bar charts illustrates the $\log_2$ fold change of taxa adjusted for false discovery rate (FDR), *p*-values <0.05 (based on DESeq2).

Supplementary Table S1: Reative abundance table of shotgun metagenomic sequencing of *ex-vivo* biofilm community exposed to low ampicillin concentrations. Untreated [Control], Ampicillin 0.025μg/mL [0.025],Ampicillin 0.050μg/mL [0.050] and Ampicillin 0.1μg/mL [0.1]

Feature.ID	DonorA-Control-1	DonorA-Control-2	DonorA-Control-3	DonorA- 0.025-1	DonorA-0.025-2	DonorA-0.025-3	DonorA-0.05-1	DonorA-0.05-2
Actinomyces odontolyticus	0.000000e+00	0.000000e+00	0.000000e+00	0.000000e+00	7.309999e-05	7.620000e-05	2.090000e-05	1.007009e-04
Alloscardovia omnicolens	0.0002218000	0.0018830006	0.0044418455	0.0000000000	0.0000000000	0.0000000000	0.0000000000	0.0000000000
Campylobacter concisus	0.000000e+00	2.860001e-05	0.000000e+00	0.000000e+00	0.000000e+00	0.000000e+00	0.000000e+00	0.000000e+00
Gemella haemolysans	1.240000e-03	2.332401e-03	1.891932e-03	5.990399e-03	5.000099e-03	5.878300e-03	3.551200e-03	5.747154e-03
Gemella morbillorum	3.120000e-05	1.097000e-04	0.000000e+00	2.382000e-04	0.000000e+00	8.470000e-05	1.417000e-04	3.700035e-05
Gemella sanguinis	9.200001e-04	2.050501e-03	7.783956e-04	3.216699e-03	2.728099e-03	1.378700e-03	9.399001e-04	3.539233e-03
Granulicatella adiacens	2.660000e-05	8.270002e-05	2.760339e-05	8.549998e-05	1.500000e-04	2.790000e-05	1.750000e-04	3.787036e-04
Granulicatella elegans	4.055000e-04	1.670401e-03	7.323899e-04	4.254999e-04	3.260999e-04	4.664000e-04	5.759001e-04	4.134039e-04
Haemophilus parainfluenzae	0.000000e+00	0.000000e+00	0.000000e+00	0.000000e+00	0.000000e+00	0.000000e+00	0.000000e+00	2.287021e-04
Haemophilus sputorum	0.000000e+00	0.000000e+00	0.000000e+00	0.000000e+00	0.000000e+00	0.000000e+00	0.000000e+00	0.000000e+00
Neisseria flavescens	0.0025174003	0.0060335018	0.0034201200	0.0104992979	0.0096590981	0.0075996000	0.0079563008	0.0097655918
Neisseria perflava	0.000000e+00	2.270001e-05	0.000000e+00	0.000000e+00	0.000000e+00	0.000000e+00	0.000000e+00	0.000000e+00
Neisseria subflava	1.050900e-03	2.737901e-03	1.277957e-03	7.380299e-03	3.328499e-03	2.455300e-03	3.115700e-03	2.025019e-03
Porphyromonas somerae	0.000000e+00	2.350001e-05	0.000000e+00	2.104000e-04	1.856000e-04	7.430000e-05	1.761000e-04	2.018019e-04
Prevotella histicola	6.370001e-05	1.093000e-04	3.090379e-05	0.000000e+00	0.000000e+00	0.000000e+00	0.000000e+00	0.000000e+00
Prevotella jejuni	3.320000e-05	5.900002e-05	4.510554e-05	0.000000e+00	0.000000e+00	0.000000e+00	0.000000e+00	0.000000e+00
Prevotella melaninogenica	0.000000e+00	3.070001e-05	0.000000e+00	0.000000e+00	0.000000e+00	0.000000e+00	0.000000e+00	0.000000e+00
Prevotella salivae	0.000000e+00	0.000000e+00	0.000000e+00	0.000000e+00	0.000000e+00	0.000000e+00	0.000000e+00	0.000000e+00
Prevotella sp oral taxon 306	0.000000e+00	0.000000e+00	0.000000e+00	0.000000e+00	0.000000e+00	0.000000e+00	0.000000e+00	0.000000e+00
Rothia mucilaginosa	3.755000e-04	9.773003e-04	4.895601e-04	2.826699e-03	2.447500e-03	3.732600e-03	2.275800e-03	1.984519e-03
Staphylococcus aureus	0.000000e+00	0.000000e+00	0.000000e+00	0.000000e+00	0.000000e+00	0.000000e+00	0.000000e+00	0.000000e+00
Streptococcus anginosus group	0.000000e+00	0.000000e+00	0.000000e+00	0.000000e+00	0.000000e+00	8.790000e-05	0.000000e+00	0.000000e+00
Streptococcus australis	1.207000e-03	2.464101e-03	1.670905e-03	1.935460e-02	2.218040e-02	2.369860e-02	1.456780e-02	1.676636e-02
Streptococcus cristatus	0.0006129001	0.0020880006	0.0008002983	0.0037265993	0.0027296995	0.0049314000	0.0033141003	0.0030437286
Streptococcus gordonii	0.000000e+00	1.270000e-05	0.000000e+00	3.084999e-04	0.000000e+00	0.000000e+00	2.478000e-04	1.307012e-04
Streptococcus infantis	0.0128731013	0.0308983093	0.0146348972	0.0950476810	0.1068360786	0.0877496000	0.0789198079	0.0808714602
Streptococcus mitis	0.100287210	0.319939896	0.130458320	0.140161072	0.109865778	0.120021500	0.202681520	0.198577767
Streptococcus oralis	0.077363408	0.094184328	0.039422441	0.031708994	0.026876195	0.019996200	0.030773903	0.039411170
Streptococcus parasanguinis	0.0657961066	0.2025907608	0.0874433380	0.2739540452	0.2333750533	0.2400055000	0.2438246244	0.2094711690
Streptococcus peroris	6.510001e-05	1.412000e-04	6.350780e-05	0.000000e+00	0.000000e+00	1.945000e-04	1.379000e-04	0.000000e+00
Streptococcus pneumoniae	0.0005090001	0.0029427009	0.0011571421	0.0008274998	0.0007091999	0.0004222000	0.0010713001	0.0011844111
Streptococcus pseudopneumoniae	2.731200e-03	7.910802e-03	4.566261e-03	8.715598e-03	6.376299e-03	6.274900e-03	7.267601e-03	7.781973e-03
Streptococcus salivarius	0.06079901	0.22023287	0.10921371	0.37158653	0.43790691	0.44296710	0.36795494	0.39023717
Streptococcus sanguinis	0.000000e+00	0.000000e+00	0.000000e+00	0.000000e+00	6.025999e-04	5.072000e-04	2.190000e-04	8.155077e-04
Streptococcus sp A12	2.786000e-04	9.205003e-04	8.930097e-04	2.508099e-03	5.374299e-03	1.013720e-02	2.191800e-03	2.371922e-03
Streptococcus sp F0442	1.617000e-04	4.507001e-04	4.077501e-04	3.390899e-03	3.819699e-03	4.062300e-03	3.954200e-03	3.253931e-03
Streptococcus sp HMSC034E03	2.800600e-03	9.719603e-03	3.234897e-03	6.053899e-03	4.028499e-03	4.256500e-03	9.659901e-03	6.211558e-03
Streptococcus sp HMSC067H01	2.869200e-03	9.103003e-03	3.977788e-03	7.286499e-03	8.690798e-03	9.759700e-03	9.189101e-03	1.026770e-02
Streptococcus sp HMSC071D03	1.755000e-04	1.004900e-03	3.021371e-04	3.956999e-04	3.013999e-04	0.000000e+00	3.048000e-04	7.117067e-04
Streptococcus sp HPH0090	2.190000e-05	0.000000e+00	2.130262e-05	0.000000e+00	1.559000e-04	7.210000e-05	2.745000e-04	1.445014e-04
Streptococcus sp oral taxon 058	0.000000e+00	0.000000e+00	1.400172e-05	1.746000e-04	0.000000e+00	0.000000e+00	0.000000e+00	5.350050e-05
Streptococcus vestibularis	0.001016100	0.003165401	0.001932037	0.003342399	0.002950599	0.002645200	0.003650800	0.003972737
Veillonella atypica	6.635093e-01	5.451462e-02	5.734878e-01	1.375000e-04	3.292099e-03	2.234000e-04	8.479001e-04	9.300087e-06
Veillonella dispar	0.000000e+00	1.866001e-04	9.621482e-03	4.039999e-04	0.000000e+00	2.130000e-04	0.000000e+00	2.397023e-04
Veillonella infantium	3.730000e-05	1.620000e-05	2.764740e-03	4.229999e-05	3.039999e-05	0.000000e+00	1.820000e-05	5.020047e-05
Veillonella parvula	0.000000e+00	1.472000e-04	0.000000e+00	0.000000e+00	0.000000e+00	0.000000e+00	0.000000e+00	0.000000e+00
Veillonella sp T11011 6	0.000000e+00	0.000000e+00	2.546313e-04	0.000000e+00	0.000000e+00	0.000000e+00	0.000000e+00	0.000000e+00
Veillonella tobetsuensis	0.000000e+00	1.921441e-02	5.217641e-04	0.000000e+00	0.000000e+00	0.000000e+00	0.000000e+00	0.000000e+00

Feature.ID	DonorA-0.05-3	DonorA-0.1-1	DonorA-0.1-2	DonorA-0.1-3	DonorB-Control-1	DonorB-Control-2	DonorB-Control-3	DonorB-0.025-1
Actinomyces odontolyticus	6.010001e-05	4.890001e-05	0.000000e+00	0.000000e+00	0.000000e+00	0.000000e+00	0.000000e+00	0.000000e+00
Alloscardovia omnicolens	0.0000000000	0.0005089002	0.0000000000	0.0000000000	0.0000000000	0.0000000000	0.0000000000	0.0000000000
Campylobacter concisus	0.000000e+00	0.000000e+00	0.000000e+00	0.000000e+00	0.000000e+00	0.000000e+00	6.400000e-06	4.009999e-05
Gemella haemolysans	3.641500e-03	4.097701e-03	4.640221e-03	2.396000e-03	2.999001e-04	0.000000e+00	0.000000e+00	0.000000e+00
Gemella morbillorum	6.110001e-05	3.017001e-04	0.000000e+00	0.000000e+00	0.000000e+00	0.000000e+00	0.000000e+00	0.000000e+00
Gemella sanguinis	2.266300e-03	1.678601e-03	1.807164e-03	1.713500e-03	2.720001e-04	0.000000e+00	0.000000e+00	0.000000e+00
Granulicatella adiacens	1.334000e-04	1.613000e-04	9.780888e-05	8.340002e-05	0.000000e+00	2.150000e-05	0.000000e+00	0.000000e+00
Granulicatella elegans	6.699001e-04	2.926001e-04	4.827438e-04	3.281001e-04	1.017000e-04	0.000000e+00	3.100000e-05	0.000000e+00
Haemophilus parainfluenzae	0.000000e+00	0.000000e+00	0.000000e+00	0.000000e+00	0.000000e+00	0.000000e+00	0.000000e+00	0.000000e+00
Haemophilus sputorum	0.000000e+00	0.000000e+00	0.000000e+00	0.000000e+00	0.000000e+00	0.000000e+00	0.000000e+00	0.000000e+00
Neisseria flavescens	0.0099974010	0.0057970017	0.0051538680	0.0045640009	0.0000000000	0.0000000000	0.0000000000	0.0000000000
Neisseria perflava	5.430001e-05	0.000000e+00	0.000000e+00	0.000000e+00	0.000000e+00	0.000000e+00	0.000000e+00	0.000000e+00
Neisseria subflava	3.135700e-03	1.988501e-03	2.144195e-03	1.190300e-03	0.000000e+00	0.000000e+00	0.000000e+00	0.000000e+00
Porphyromonas somerae	1.608000e-04	3.360001e-04	4.000363e-06	3.810001e-05	0.000000e+00	0.000000e+00	0.000000e+00	0.000000e+00
Prevotella histicola	0.000000e+00	0.000000e+00	0.000000e+00	0.000000e+00	7.680002e-05	8.410001e-05	1.484000e-04	4.914999e-04
Prevotella jejuni	0.000000e+00	0.000000e+00	0.000000e+00	0.000000e+00	0.000000e+00	0.000000e+00	0.000000e+00	0.000000e+00
Prevotella melaninogenica	0.000000e+00	0.000000e+00	0.000000e+00	0.000000e+00	3.800001e-05	1.790000e-05	2.570000e-05	1.451300e-03
Prevotella salivae	0.000000e+00	0.000000e+00	0.000000e+00	0.000000e+00	0.000000e+00	0.000000e+00	0.000000e+00	2.534999e-04
Prevotella sp oral taxon 306	0.000000e+00	0.000000e+00	0.000000e+00	0.000000e+00	0.000000e+00	0.000000e+00	0.000000e+00	1.114000e-04
Rothia mucilaginosa	1.966600e-03	1.863901e-03	2.388217e-03	2.242200e-03	0.000000e+00	0.000000e+00	0.000000e+00	0.000000e+00
Staphylococcus aureus	0.000000e+00	0.000000e+00	0.000000e+00	0.000000e+00	3.860001e-04	7.900001e-05	3.457000e-04	2.999999e-05
Streptococcus anginosus group	1.440000e-05	0.000000e+00	6.980634e-05	0.000000e+00	0.000000e+00	0.000000e+00	0.000000e+00	0.000000e+00
Streptococcus australis	1.782700e-02	2.763601e-02	1.694474e-02	1.956860e-02	9.070002e-05	3.000000e-05	3.810000e-05	0.000000e+00
Streptococcus cristatus	0.0025470003	0.0032589010	0.0026742428	0.0038143008	0.0162476032	0.0227275023	0.0235364000	0.0036860993
Streptococcus gordonii	0.000000e+00	4.111001e-04	1.151105e-04	0.000000e+00	1.790000e-04	2.127000e-04	2.878000e-04	3.509999e-05
Streptococcus infantis	0.0908368091	0.0842533253	0.0621536436	0.0773037155	0.0187684038	0.0059419006	0.0134697000	0.0017182997
Streptococcus mitis	0.118751512	0.105485132	0.146477400	0.126932525	0.192413338	0.044563204	0.085596800	0.005129999
Streptococcus oralis	0.025226003	0.037390511	0.035148591	0.031092306	0.036825107	0.012128201	0.026117300	0.001172300
Streptococcus parasanguinis	0.2360467236	0.2552650766	0.2329976562	0.2273580455	0.0132793027	0.0073654007	0.0084863000	0.0007302999
Streptococcus peroris	8.730001e-05	0.000000e+00	0.000000e+00	1.316000e-04	0.000000e+00	0.000000e+00	0.000000e+00	0.000000e+00
Streptococcus pneumoniae	0.0002788000	0.0005390002	0.0006944631	0.0004205001	0.0000000000	0.0000000000	0.0000000000	0.0000000000
Streptococcus pseudopneumoniae	2.885000e-03	4.338201e-03	9.348149e-03	7.322901e-03	5.107001e-04	2.740000e-05	8.880000e-05	1.750000e-05
Streptococcus salivarius	0.45596955	0.43505723	0.44693278	0.46049869	0.10096482	0.03296760	0.06163130	0.01911020
Streptococcus sanguinis	2.770000e-04	1.260300e-03	2.555232e-04	7.204001e-04	1.732000e-04	0.000000e+00	9.500000e-05	0.000000e+00
Streptococcus sp A12	5.197201e-03	3.993801e-03	3.449713e-03	3.632801e-03	3.456001e-04	4.440000e-05	1.578000e-04	0.000000e+00
Streptococcus sp F0442	3.875400e-03	5.935602e-03	4.531711e-03	5.724401e-03	0.000000e+00	0.000000e+00	6.920000e-05	0.000000e+00
Streptococcus sp HMSC034E03	5.058901e-03	5.341102e-03	4.885544e-03	5.008001e-03	2.573601e-03	7.516001e-04	1.439200e-03	5.099999e-05
Streptococcus sp HMSC067H01	8.739901e-03	1.015180e-02	7.126047e-03	1.154030e-02	2.134200e-03	5.530001e-04	1.138200e-03	6.309999e-05
Streptococcus sp HMSC071D03	1.583000e-04	1.207000e-04	4.057368e-04	0.000000e+00	4.547001e-04	1.230000e-04	1.718000e-04	0.000000e+00
Streptococcus sp HPH0090	0.000000e+00	1.023000e-04	2.636239e-04	1.437000e-04	0.000000e+00	0.000000e+00	0.000000e+00	0.000000e+00
Streptococcus sp oral taxon 058	0.000000e+00	0.000000e+00	4.850440e-05	0.000000e+00	0.000000e+00	0.000000e+00	0.000000e+00	0.000000e+00
Streptococcus vestibularis	0.003963900	0.002195301	0.008568178	0.006098201	0.066295713	0.039162504	0.058833600	0.013659697
Veillonella atypica	4.810000e-05	1.622000e-04	1.026093e-04	0.000000e+00	5.475326e-01	8.331947e-01	7.182699e-01	9.445142e-01
Veillonella dispar	6.410001e-05	0.000000e+00	8.800799e-05	1.205000e-04	0.000000e+00	0.000000e+00	0.000000e+00	6.906199e-03
Veillonella infantium	0.000000e+00	2.730001e-05	0.000000e+00	1.290000e-05	1.650000e-05	4.400000e-06	1.560000e-05	5.412999e-04
Veillonella parvula	0.000000e+00	0.000000e+00	0.000000e+00	0.000000e+00	0.000000e+00	0.000000e+00	0.000000e+00	1.852000e-04
Veillonella sp T11011 6	0.000000e+00	0.000000e+00	0.000000e+00	0.000000e+00	0.000000e+00	0.000000e+00	0.000000e+00	1.017000e-04
Veillonella tobetsuensis	0.000000e+00	0.000000e+00	0.000000e+00	0.000000e+00	2.050000e-05	0.000000e+00	0.000000e+00	0.000000e+00

Feature.ID	DonorB-0.025-2	DonorB-0.025-3	DonorB-0.05-1	DonorB-0.05-2	DonorB-0.05-3	DonorB-0.1-1	DonorB-0.1-2	DonorB-0.1-3
Actinomyces odontolyticus	0.000000e+00	0.000000e+00	0.000000e+00	0.000000e+00	0.000000e+00	0.000000e+00	0.000000e+00	0.000000e+00
Alloscardovia omnicolens	0.0000000000	0.0000000000	0.0000000000	0.0000000000	0.0000000000	0.0000000000	0.0000000000	0.0000000000
Campylobacter concisus	0.000000e+00	0.000000e+00	0.000000e+00	0.000000e+00	0.000000e+00	0.000000e+00	0.000000e+00	0.000000e+00
Gemella haemolysans	0.000000e+00	0.000000e+00	0.000000e+00	0.000000e+00	0.000000e+00	0.000000e+00	0.000000e+00	0.000000e+00
Gemella morbillorum	0.000000e+00	0.000000e+00	0.000000e+00	0.000000e+00	0.000000e+00	0.000000e+00	0.000000e+00	0.000000e+00
Gemella sanguinis	3.540001e-05	0.000000e+00	0.000000e+00	0.000000e+00	2.370000e-05	9.800000e-06	2.920000e-05	1.416000e-04
Granulicatella adiacens	0.000000e+00	0.000000e+00	0.000000e+00	0.000000e+00	0.000000e+00	0.000000e+00	0.000000e+00	0.000000e+00
Granulicatella elegans	0.000000e+00	0.000000e+00	0.000000e+00	0.000000e+00	0.000000e+00	0.000000e+00	0.000000e+00	0.000000e+00
Haemophilus parainfluenzae	0.000000e+00	0.000000e+00	0.000000e+00	0.000000e+00	0.000000e+00	0.000000e+00	0.000000e+00	0.000000e+00
Haemophilus sputorum	0.000000e+00	0.000000e+00	0.000000e+00	0.000000e+00	0.000000e+00	0.000000e+00	0.000000e+00	0.000000e+00
Neisseria flavescens	0.0000000000	0.0000000000	0.0000000000	0.0000000000	0.0000000000	0.0000000000	0.0000000000	0.0000000000
Neisseria perflava	0.000000e+00	0.000000e+00	0.000000e+00	0.000000e+00	0.000000e+00	0.000000e+00	0.000000e+00	0.000000e+00
Neisseria subflava	0.000000e+00	0.000000e+00	0.000000e+00	0.000000e+00	0.000000e+00	0.000000e+00	0.000000e+00	0.000000e+00
Porphyromonas somerae	0.000000e+00	0.000000e+00	0.000000e+00	0.000000e+00	0.000000e+00	0.000000e+00	0.000000e+00	0.000000e+00
Prevotella histicola	6.271002e-04	3.548000e-04	2.973999e-04	1.792000e-04	2.446000e-04	1.802000e-04	9.839999e-05	1.392000e-04
Prevotella jejuni	0.000000e+00	0.000000e+00	0.000000e+00	0.000000e+00	0.000000e+00	0.000000e+00	0.000000e+00	0.000000e+00
Prevotella melaninogenica	2.610301e-03	1.777200e-03	5.048998e-04	1.614000e-04	5.181999e-04	1.747000e-04	1.103000e-04	5.300001e-06
Prevotella salivae	5.987002e-04	4.871000e-04	2.387999e-04	1.088000e-04	4.005999e-04	2.144000e-04	7.229999e-05	4.100000e-06
Prevotella sp oral taxon 306	7.540002e-05	1.499000e-04	4.529999e-05	0.000000e+00	4.499999e-06	0.000000e+00	0.000000e+00	0.000000e+00
Rothia mucilaginosa	0.000000e+00	0.000000e+00	0.000000e+00	0.000000e+00	0.000000e+00	0.000000e+00	0.000000e+00	0.000000e+00
Staphylococcus aureus	1.372000e-04	7.790001e-05	4.179999e-05	8.470000e-05	1.660000e-05	3.000000e-05	2.390000e-05	6.060001e-04
Streptococcus anginosus group	0.000000e+00	0.000000e+00	0.000000e+00	0.000000e+00	0.000000e+00	1.309000e-04	0.000000e+00	0.000000e+00
Streptococcus australis	5.060002e-05	1.260000e-05	2.159999e-05	0.000000e+00	4.739999e-05	3.400000e-06	0.000000e+00	4.530000e-05
Streptococcus cristatus	0.0244060073	0.0023189002	0.0108771967	0.0057604000	0.0056493989	0.0116412000	0.0101510990	0.0264914026
Streptococcus gordonii	1.216000e-04	7.040001e-05	8.989997e-05	4.770000e-05	0.000000e+00	7.110000e-05	1.494000e-04	0.000000e+00
Streptococcus infantis	0.0030493009	0.0016063002	0.0027125992	0.0023279000	0.0032609993	0.0043231000	0.0031776997	0.0108455011
Streptococcus mitis	0.010425503	0.009309801	0.009892297	0.007004000	0.012895197	0.031542700	0.010512399	0.108671011
Streptococcus oralis	0.001404300	0.002935500	0.001126300	0.003953600	0.001862900	0.010548700	0.001241400	0.013504201
Streptococcus parasanguinis	0.0020252006	0.0013918001	0.0013382996	0.0011878000	0.0021949996	0.0037816000	0.0021221998	0.0124767012
Streptococcus peroris	0.000000e+00	0.000000e+00	0.000000e+00	0.000000e+00	0.000000e+00	0.000000e+00	0.000000e+00	0.000000e+00
Streptococcus pneumoniae	0.0000000000	0.0000000000	0.0000000000	0.0000000000	0.0000000000	0.0000000000	0.0000000000	0.0000000000
Streptococcus pseudopneumoniae	0.000000e+00	6.790001e-05	0.000000e+00	0.000000e+00	6.549999e-05	3.010000e-05	0.000000e+00	2.765000e-04
Streptococcus salivarius	0.07097882	0.03068300	0.04546829	0.02276230	0.05785039	0.05060480	0.04400560	0.14949581
Streptococcus sanguinis	6.710002e-05	5.010001e-05	1.320000e-05	0.000000e+00	0.000000e+00	0.000000e+00	0.000000e+00	9.020001e-05
Streptococcus sp A12	6.540002e-05	2.020000e-05	0.000000e+00	2.800000e-06	2.710999e-04	1.450000e-05	0.000000e+00	1.189000e-04
Streptococcus sp F0442	0.000000e+00	1.000000e-05	0.000000e+00	0.000000e+00	0.000000e+00	0.000000e+00	0.000000e+00	8.480001e-05
Streptococcus sp HMSC034E03	1.748001e-04	1.346000e-04	1.894999e-04	2.011000e-04	1.564000e-04	4.266000e-04	1.995000e-04	1.854600e-03
Streptococcus sp HMSC067H01	1.031000e-04	1.576000e-04	1.626000e-04	1.152000e-04	2.028000e-04	4.758000e-04	4.076000e-04	1.449500e-03
Streptococcus sp HMSC071D03	3.310001e-05	0.000000e+00	0.000000e+00	6.370000e-05	0.000000e+00	3.250000e-05	3.450000e-05	4.758000e-04
Streptococcus sp HPH0090	0.000000e+00	0.000000e+00	0.000000e+00	0.000000e+00	0.000000e+00	0.000000e+00	0.000000e+00	0.000000e+00
Streptococcus sp oral taxon 058	0.000000e+00	0.000000e+00	0.000000e+00	0.000000e+00	0.000000e+00	0.000000e+00	0.000000e+00	0.000000e+00
Streptococcus vestibularis	0.017606805	0.012591501	0.013905796	0.008005200	0.019351096	0.024578100	0.013935199	0.078318508
Veillonella atypica	8.653989e-01	9.357340e-01	8.860221e-01	9.466408e-01	8.949579e-01	8.611425e-01	8.854949e-01	4.677416e-01
Veillonella dispar	0.000000e+00	0.000000e+00	3.241999e-03	3.690000e-05	0.000000e+00	4.330000e-05	5.766399e-03	2.782000e-04
Veillonella infantium	5.400002e-06	5.890001e-05	3.912999e-04	1.110000e-05	2.569999e-05	0.000000e+00	1.259100e-03	1.578000e-04
Veillonella parvula	0.000000e+00	0.000000e+00	2.339179e-02	1.345400e-03	0.000000e+00	0.000000e+00	2.742000e-03	5.929001e-04
Veillonella sp T11011 6	0.000000e+00	0.000000e+00	2.699999e-05	0.000000e+00	0.000000e+00	0.000000e+00	9.049999e-05	0.000000e+00
Veillonella tobetsuensis	0.000000e+00	0.000000e+00	0.000000e+00	0.000000e+00	0.000000e+00	0.000000e+00	1.837640e-02	1.261345e-01

Supplementary Table S2: Total ARG abundance, RPKM (reads per kilobase per million). Shotgun metagenomic sequencing of *ex-vivo* biofilm community exposed to low ampicillin concentrations. Untreated [Control], Ampicillin 0.025µg/mL [0.025],Ampicillin 0.050µg/mL [0.050] and Ampicillin 0.1µg/mL [0.1]

Feature.ID	DonorA-Control-1	DonorA-Control-2	DonorA-Control-3	DonorA- 0.025-1	DonorA-0.025-2	DonorA-0.025-3	DonorA-0.05-1	DonorA-0.05-2	DonorA-0.05-3	DonorA-0.1-1	DonorA-0.1-2	DonorA-0.1-3
patB	185.506091830803	329.121435013339	198.756382941826	92.1846232732832	104.937773636337	108.61913023648	161.337391191856	170.831280741016	116.704349815948	89.6226967225611	136.330406572138	127.428784315637
patA	175.75992960229	318.340479452363	203.98966931559	96.7285376670302	108.399411728267	98.4596669806911	154.399798228685	158.615812781967	117.208401967705	92.0063135313078	131.914670387693	128.019353163302
pmrA	83.6731183379788	188.983023991182	106.800768965537	50.0635236406296	58.432262399813	55.8546298165059	75.738839187319	82.8564788265597	62.9028732459927	45.9198899132161	75.6895217129974	67.9869658319358
mefE	0	0	0	0	0	0	0	0	0	0	0	0
CfxA2	0	0	0	0	0	0	0	0	0	0	0	0
mel	60.9041098879392	162.710156619149	68.5062403713675	69.9272263858641	91.3637089520886	83.097773059899	95.4217575613705	97.1003029125243	97.8661287365814	86.5209593147135	78.9828033144109	93.8257734762391
ErmB	1.70432774217028	2.48333077410576	0	0	2.95507499865186	0	0	0	3.36829307876802	3.74647959961251	0	0
CfxA3	0	0	0	0	0	0	0	0	0	0	0	0
tetM	57.1141530498038	112.196593358773	59.375427503078	38.4190015280148	36.1820143332176	33.9719854169185	62.4913533762277	59.1627347084529	47.4501708774025	39.1042845364296	45.4628620678718	69.8023077834974
aad(6)	0	0	0	0	0	0	0	0	0	0	0	0
CfxA4	0	0	0	0	0	0	0	0	0	0	0	0
CfxA5	0	0	0	0	0	0	0	0	0	0	0	0
lnuC	0	0	0	0	0	0	0	0	0	0	0	0
mgrA	0	0	0	0	0	0	0	0	0	0	0	0
PC1	0	0	0	0	0	0	0	0	0	0	0	0
APH(3')-III	0	0	0	0	0	0	0	0	0	0	0	0
RlmA(II)	218.536973062821	372.721185381045	233.122997684641	141.343186124996	182.615158501182	182.972139318741	205.823573458179	236.005043082753	180.163510141153	150.269119244699	195.747099530552	189.464501627069
lsaC	9.12454897096194	12.0199757803446	9.08728652379765	10.797586427229	16.9445220391615	15.0046697847591	15.2523236211275	10.2280756829882	19.2805741750169	15.0726558082193	12.2291053056132	15.6413530443837
tetB(46)	52.2837121872686	100.566318352064	72.0788148799599	96.928918336157	125.139905657631	131.126616810241	105.118330254638	109.65271183424	131.032563631789	141.561638671512	121.55686595004	135.755958558403
tetA(46)	56.9773066472885	114.88751928239	79.3512959670121	105.538426501413	135.118725935799	156.464676462929	130.677334049813	128.353051570881	148.718194041492	158.378689827841	141.321297834168	150.200512991077
tetA(60)	0	3.68699691871004	3.40232334730281	6.64308650665709	11.6416767999627	7.23873934210676	9.95821998741728	11.4600938902572	10.3633086190716	13.2554546642414	7.83371673200152	8.68616472398111
tetB(60)	0	16.1250588131536	3.31036866224057	4.54526971508117	7.23873493331017	0	0	7.98254815804124	5.60341686961429	7.15461778948593	4.67010035946245	0
tetO	15.3836882827645	34.9431801347023	22.3334941344911	6.33715489121893	7.91270219997468	11.7925742481717	11.6639174557809	13.966989428751	9.5555580017784	7.43886132569612	10.853746349537	11.4864557112084
SAT-4	0	0	0	0	0	0	0	0	0	0	0	0
TEM-116	0	0	0	0	0	0	2.65796943980765	2.87500613274746	2.43526415973646	0	0	0
CfxA	0	0	0	0	0	0	0	0	0	0	0	0
tetQ	0	0	0	0	0	0	0	0	0	0	0	0

Feature.ID	DonorB-Control-1	DonorB-Control-2	DonorB-Control-3	DonorB-0.025-1	DonorB-0.025-2	DonorB-0.025-3	DonorB-0.05-1	DonorB-0.05-2	DonorB-0.05-3	DonorB-0.1-1	DonorB-0.1-2	DonorB-0.1-3
patB	267.950466165674	87.4260118219767	149.83787840089	10.5268602807948	15.4459323360565	17.9451748030036	23.2597307545428	16.3307006213872	28.0134214278123	55.6626457422054	18.4209948932731	127.294024048512
patA	270.36598071766	85.9911673674018	145.073729595421	10.4911621138957	19.3615655572597	18.9139316853537	21.8799269994219	17.2125089510337	26.1253816998501	58.9244026403186	25.4815402810589	127.386614645015
pmrA	174.468402531637	50.209667525662	87.3562456414511	6.63434315476534	11.8785160407481	12.8329787581302	15.9183962046479	9.16707187781489	17.8148076936046	31.0953345100889	12.9104162646833	80.6120975891439
mefE	11.5160661737054	0	4.44685513204465	0	0	0	0	0	0	0	0	6.97514532743877
CfxA2	0	0	0	0	0	0	0	0	0	0	0	0
mel	195.318029179735	59.9861983084184	100.667599799562	17.8984627258988	43.9995438940147	36.6656535946578	43.1703701703453	38.4824606564943	57.8782750542868	64.102076388864	50.236082594329	170.447474400522
ErmB	14.9477613949462	30.5730242048325	27.9580389928349	3.98414181872332	31.0636451985566	3.65451749957707	20.1989390446058	8.3235155866313	7.15454124241151	13.3025265496277	12.3599782502343	28.3855212969679
CfxA3	0	0	0	1.23236187933181	2.05896565779253	1.23185002195491	0.42073202602479	0	0	0	0	0
tetM	93.4536767264372	55.8959703964419	59.4398618720349	8.29292894345667	34.8758755847545	8.09352637586623	20.8770189585559	13.119541274409	19.2861199361568	29.5743670612259	17.4421027818322	50.3103796737404
aad(6)	0	0	0	0	0	0	0	0	0	0	0	0
CfxA4	0	0	0	0	0	0	0	0	0	0	0	0
CfxA5	0	0	0	0	0	0	0	0	0	0	0	0
lnuC	0	122.095507528434	13.8822732031285	38.6298768796305	8.70588913486821	0	0	117.396493613123	0	44.6560139868208	67.0219344502687	5.31954451400099
mgrA	1.39714436461772	0	0	0	0	0	0	0	0	0	0	0
PC1	1.46650614158455	1.42658247349136	0	0	0	0	0.600512909485737	0	0	0	0	2.62105216815278
APH(3')-III	0	0.81743796884526[illegible]	0	0	0	0	0	0	0	0	0	2.09189635307288
RlmA(II)	268.335645406596	87.6981784546618	139.500831013354	12.3568228898902	24.0778532066551	18.8805148449202	26.0300065747757	21.5949867557477	30.5756416750408	62.8212703652896	23.0403259890125	130.752761891936
lsaC	0	0	0	0	0	0	0	0	0	0	0	0
tetB(46)	11.9638026939113	6.57399225129819	6.59348036418019	1.11370181927525	2.29011033440136	1.45073711820596	2.01809675177435	1.60620517996306	0	3.38576519033033	2.072117193264	5.10629852448418
tetA(46)	11.237900324099	7.74992121518894	9.06075734383219	0	3.26689217703081	1.29851484667247	1.76707450930412	1.24769220265155	2.06548494998315	3.23297428743836	2.26796947996192	8.75716321137829
tetA(60)	1.69343532470044	0	0	0	0	0	0	0	0	0	0	0
tetB(60)	0	0	0	0	0	0	0	0	0	0	0	0
tetO	5.08866174050609	0.87035248022141[illegible]	0	0	0	1.1666344325573	0	0	0	0	0	0
SAT-4	1.4280204831728	0	0.992557720080684	0	0	0	0	0	0	0	0	2.55226764992778
TEM-116	0	0	0	0	0	0	0	0	0	0	0	0
CfxA	0	0	0	0	0	0	0	0	0	0	0	0
tetQ	0	0	0	1.73382828235778	2.6309005627349	2.30490085459648	0.849296829129828	0	0	0	0	0

Supplementary Table S3: Relative abundance table of antimicrobial resistance genes (ARGs). Shotgun metagenomic sequencing of *ex-vivo* biofilm exposed to low ampicillin concentrations. Untreated [Control], Ampicillin 0.025µg/mL [0.025],Ampicillin 0.050µg/mL [0.050] and Ampicillin 0.1µg/mL [0.1]

Gene.ID	DonorA-Control-1	DonorA-Control-2	DonorA-Control-3	DonorA- 0.025-1	DonorA-0.025-2	DonorA-0.025-3	DonorA-0.05-1	DonorA-0.05-2	DonorA-0.05-3	DonorA-0.1-1	DonorA-0.1-2	DonorA-0.1-3
aad(6)	0	0	0	0	0	0	0	0	0	0	0	0
APH(3)-IIIa	0	0,000893997	0	0	0	0	0	0	0	0	0	0,001639568
CfxA	0	0	0	0	0	0	0	0	0	0	0	0
CfxA2	0	0	0	0	0	0	0	0	0	0	0	0
CfxA3	0	0	0	0,009821977	0,007659574	0,006964359	0,001718951	0	0	0	0	0
CfxA4	0	0	0	0	0	0	0	0	0	0	0	0
CfxA5	0	0	0	0	0	0	0	0	0	0	0	0
ErmB	0,00598106	0,031417625	0,020248204	0,024554942	0,089361702	0,015977059	0,063816072	0,027187173	0,020750988	0,021267361	0,036151961	0,020904495
lnuC	0	0,083141762	0,006662312	0,1577655	0,016595745	0	0	0,254095504	0	0,047309028	0,129901961	0,002595983
lsaC	0	0	0	0	0	0	0	0	0	0	0	0
mefE	0,007476325	0	0,005225343	0	0	0	0	0	0	0	0	0,008334472
mel	0,127429806	0,100510856	0,118876551	0,179864948	0,206382979	0,261368292	0,222389343	0,20494946	0,273715415	0,167100694	0,239583333	0,20467277
mgrA	0,000332281	0	0	0	0	0	0	0	0	0	0	0
patA	0,24547267	0,200510856	0,23840627	0,146715777	0,126382979	0,187628021	0,156854319	0,127570582	0,171936759	0,213758681	0,169117647	0,212870611
patB	0,253613557	0,212515964	0,256694971	0,153468386	0,105106383	0,18557968	0,173828964	0,126176368	0,192193676	0,210503472	0,12745098	0,221751605
PC1 beta-lactamase (blaZ)	0,000664562	0,001660281	0	0	0	0	0,002148689	0	0	0	0	0,002186091
pmrA	0,112144875	0,082886335	0,10163292	0,065684469	0,054893617	0,090126997	0,080790718	0,048100383	0,083003953	0,079861111	0,060661765	0,09536822
RlmA(II)	0,122030238	0,102426564	0,114826911	0,086556169	0,078723404	0,093814011	0,093467985	0,080167306	0,100790514	0,114149306	0,076593137	0,10944118
SAT-4	0,000415351	0	0,000522534	0	0	0	0	0	0	0	0	0,001366307
Staphylococcus aureus FosB	0	0	0	0	0	0	0,000644607	0	0	0	0	0
tetA(46)	0,010383785	0,018390805	0,015153494	0	0,021702128	0,013109381	0,012892136	0,009410945	0,013833992	0,011935764	0,015318627	0,014892745
tetA(60)	0,001578335	0	0	0	0	0	0	0	0	0	0	0
tetB(46)	0,011131417	0,015708812	0,011103854	0,015960712	0,015319149	0,014748054	0,014825956	0,012199373	0	0,012586806	0,014093137	0,008744364
tetB(60)	0	0	0	0	0	0	0	0	0	0	0	0
tetM	0,096112311	0,147637292	0,110646636	0,131368938	0,25787234	0,090946333	0,169531586	0,110142907	0,143774704	0,121527778	0,131127451	0,095231589
tetO	0,005233427	0,002298851	0	0	0	0,013109381	0	0	0	0	0	0
tetQ	0	0	0	0,028238183	0,02	0,026628431	0,007090675	0	0	0	0	0

Gene.ID	DonorB-Control-1	DonorB-Control-2	DonorB-Control-3	DonorB- 0.025-1	DonorB-0.025-2	DonorB-0.025-3	DonorB-0.05-1	DonorB-0.05-2	DonorB-0.05-3	DonorB-0.1-1	DonorB-0.1-2	DonorB-0.1-3
aad(6)	0	0	0	0	0	0	0	0	0	0	0	0,003499
APH(3)-IIIa	0,000841815	0,001530724	0	0,001567574	0,003642384	0,002375767	0	0,006935414	0	0,006849315	0	0
CfxA	0	0	0	0	0	0	0	0,027741656	0	0	0,012408635	0
CfxA2	0	0	0	0	0	0	0	0,039011704	0	0	0,017678055	0
CfxA3	0	0	0	0	0	0	0	0,018205462	0	0	0,009688934	0
CfxA4	0	0	0	0	0	0	0	0,015604681	0	0	0,006459289	0
CfxA5	0	0	0	0	0	0	0	0,022973559	0	0	0,006969233	0
ErmB	0,005280478	0,022195495	0,013302752	0,00481469	0,004470199	0,012274797	0,012311358	0,007368877	0,016049383	0,021481943	0,004929458	0,018894
lnuC	0	0	0	0	0	0	0	0	0	0	0	0
lsaC	0,010025254	0,014104527	0,019877676	0,014556041	0,010430464	0,027123342	0,021577972	0,022540095	0	0,077833126	0,010538841	0,031491
mefE	0	0	0	0	0	0	0	0	0	0	0	0
mel	0,175556746	0,176361251	0,198929664	0,191020043	0,22102649	0,193823005	0,268599418	0,189423494	0,209876543	0,210772105	0,110317865	0,249825
mgrA	0	0	0	0	0	0	0	0	0	0	0	0
patA	0,182903497	0,166192871	0,159480122	0,175680215	0,163245033	0,150069293	0,171035213	0,11053316	0,154320988	0,146637609	0,070882203	0,145906
patB	0,198285758	0,177782637	0,174923547	0,192587616	0,177980132	0,165907741	0,157797194	0,110099697	0,193209877	0,137297634	0,064252932	0,145906
PC1 beta-lactamase (blaZ)	0	0	0	0	0	0	0	0	0	0	0	0
pmrA	0,070942068	0,055762082	0,064525994	0,080170194	0,07384106	0,048703227	0,051098756	0,045513654	0,058641975	0,063200498	0,023967364	0,048286
RlmA(II)	0,075074615	0,080581675	0,085779817	0,09103124	0,068708609	0,077410414	0,066851999	0,044646727	0,077777778	0,082191781	0,026857046	0,060182
SAT-4	0,001683631	0	0,001376147	0,001343635	0,002483444	0	0	0	0	0,002801993	0	0
Staphylococcus aureus FosB	0	0	0	0	0	0	0	0	0	0	0	0
tetA(46)	0,016071019	0,020883446	0,012691131	0,009629381	0,014900662	0,019006137	0,014826582	0,009969658	0	0,014009963	0,007139215	0,016445
tetA(60)	0,007346751	0,006669582	0,005504587	0,00727802	0,009437086	0,007325282	0,007678051	0,015604681	0	0,01992528	0	0,012946
tetB(46)	0,014999617	0,015416576	0,015749235	0,015563767	0,01705298	0,016036428	0,015753243	0,011270048	0,014197531	0,012764633	0,011048785	0,017495
tetB(60)	0,00742328	0,006669582	0,009480122	0,009181503	0,004635762	0,005741437	0,003839026	0	0	0,019613948	0	0,006998
tetM	0,22560649	0,251585393	0,227217125	0,198298063	0,217384106	0,260740447	0,200423617	0,238838318	0,259876543	0,174968867	0,123746388	0,222883
tetO	0,007958981	0,004264159	0,01116208	0,00727802	0,010761589	0,013462681	0,008207572	0	0,016049383	0,009651308	0,003059663	0,019244
tetQ	0	0	0	0	0	0	0	0,063719116	0	0	0,490056094	0

Supplementary Table S4: Reative abundance table of shotgun metagenomic sequencing of *ex-vivo* biofilm community exposed to high ampicillin concentrations. Untreated [Control], Ampicillin 10 µg/mL [10.0]

Feature.ID	DonorC-Control-1	DonorC-Control-2	DonorC-Control-3	DonorC-0.10-1	DonorC-0.10-2	DonorC-0.10-3
Actinomyces odontolyticus	0.000000e+00	0.000000e+00	0.000000e+00	0.000000e+00	0.000000e+00	0.000000e+00
Alloscardovia omnicolens	0.0000000000	0.0000000000	0.0000000000	0.0000000000	0.0000000000	0.0000000000
Campylobacter concisus	9.230000e-05	1.940000e-05	3.639999e-05	0.000000e+00	0.000000e+00	0.000000e+00
Gemella haemolysans	1.641000e-04	2.303000e-04	1.300000e-05	5.409999e-05	1.171000e-04	3.096000e-04
Gemella morbillorum	1.650000e-05	2.340000e-05	1.350000e-05	0.000000e+00	0.000000e+00	1.090000e-05
Gemella sanguinis	4.884000e-04	3.348000e-04	2.269999e-04	2.439000e-04	2.066000e-04	1.675000e-04
Granulicatella adiacens	0.000000e+00	0.000000e+00	0.000000e+00	0.000000e+00	0.000000e+00	0.000000e+00
Granulicatella elegans	2.611000e-04	1.239000e-04	3.409999e-05	1.110000e-04	2.320000e-05	3.760000e-05
Haemophilus parainfluenzae	7.480000e-05	1.690000e-05	3.739999e-05	5.899999e-06	0.000000e+00	0.000000e+00
Haemophilus sputorum	2.784000e-04	1.378000e-04	1.268000e-04	3.250000e-05	1.059000e-04	4.640000e-05
Neisseria flavescens	0.0037186000	0.0014754000	0.0014628996	0.0013601999	0.0012808003	0.0013984999
Neisseria perflava	0.000000e+00	0.000000e+00	0.000000e+00	0.000000e+00	0.000000e+00	0.000000e+00
Neisseria subflava	0.000000e+00	1.800000e-05	7.869998e-05	0.000000e+00	0.000000e+00	4.200000e-05
Porphyromonas somerae	0.000000e+00	0.000000e+00	0.000000e+00	0.000000e+00	0.000000e+00	0.000000e+00
Prevotella histicola	0.000000e+00	0.000000e+00	0.000000e+00	0.000000e+00	0.000000e+00	0.000000e+00
Prevotella jejuni	6.190000e-05	1.870000e-05	3.419999e-05	7.539999e-05	1.381000e-04	3.110000e-05
Prevotella melaninogenica	1.110000e-05	4.250000e-05	3.939999e-05	2.040000e-05	2.420000e-05	2.291000e-04
Prevotella salivae	0.000000e+00	0.000000e+00	0.000000e+00	0.000000e+00	0.000000e+00	0.000000e+00
Prevotella sp oral taxon 306	0.000000e+00	0.000000e+00	0.000000e+00	0.000000e+00	0.000000e+00	0.000000e+00
Rothia mucilaginosa	3.086000e-04	4.678000e-04	1.832999e-04	1.381000e-04	1.102000e-04	1.277000e-04
Staphylococcus aureus	0.000000e+00	0.000000e+00	0.000000e+00	0.000000e+00	0.000000e+00	0.000000e+00
Streptococcus anginosus grou	0.000000e+00	0.000000e+00	0.000000e+00	0.000000e+00	0.000000e+00	0.000000e+00
Streptococcus australis	4.940000e-05	3.600000e-06	3.869999e-05	5.749999e-05	0.000000e+00	0.000000e+00
Streptococcus cristatus	0.0000000000	0.0000000000	0.0000000000	0.0000000000	0.0000000000	0.0000000000
Streptococcus gordonii	0.000000e+00	0.000000e+00	0.000000e+00	0.000000e+00	0.000000e+00	0.000000e+00
Streptococcus infantis	0.0078342000	0.0153985000	0.0038168989	0.0031003997	0.0029855006	0.0032928997
Streptococcus mitis	0.067872700	0.036007800	0.024458193	0.032634297	0.020367704	0.019988798
Streptococcus oralis	0.068925400	0.047951800	0.031521291	0.037331296	0.021755604	0.040139996
Streptococcus parasanguinis	0.0122011000	0.0096592000	0.0057256983	0.0048244995	0.0039719008	0.0056831994
Streptococcus peroris	0.000000e+00	0.000000e+00	0.000000e+00	0.000000e+00	0.000000e+00	0.000000e+00
Streptococcus pneumoniae	0.0016724000	0.0005060000	0.0005777998	0.0008539999	0.0003944001	0.0005571999
Streptococcus pseudopneum	0.000000e+00	0.000000e+00	0.000000e+00	0.000000e+00	2.860001e-05	0.000000e+00
Streptococcus salivarius	0.05054790	0.05085230	0.04550859	0.06223939	0.05344511	0.04796000
Streptococcus sanguinis	4.790000e-05	0.000000e+00	8.289998e-05	5.269999e-05	5.830001e-05	0.000000e+00
Streptococcus sp A12	2.552000e-04	1.259000e-04	1.005000e-04	1.320000e-05	1.450000e-05	9.899999e-06
Streptococcus sp F0442	1.010000e-05	0.000000e+00	0.000000e+00	8.019999e-05	6.700001e-06	0.000000e+00
Streptococcus sp HMSC034E0	1.364800e-03	6.610000e-04	3.376999e-04	4.813000e-04	2.885001e-04	3.247000e-04
Streptococcus sp HMSC067H0	3.247000e-04	1.004000e-04	2.783999e-04	1.380000e-04	1.513000e-04	1.265000e-04
Streptococcus sp HMSC071D0	2.294700e-03	1.236100e-03	1.412600e-03	2.493300e-03	1.870200e-03	1.957700e-03
Streptococcus sp HPH0090	0.000000e+00	0.000000e+00	5.199998e-05	0.000000e+00	2.020000e-05	0.000000e+00
Streptococcus sp oral taxon 0	0.000000e+00	0.000000e+00	0.000000e+00	0.000000e+00	0.000000e+00	0.000000e+00
Streptococcus vestibularis	0.000000000	0.000000000	0.000000000	0.000000000	0.000000000	0.000135000
Veillonella atypica	7.798714e-01	8.343972e-01	8.754070e-01	8.525568e-01	8.916748e-01	8.735733e-01
Veillonella dispar	8.062000e-04	1.148000e-04	3.467999e-04	7.393999e-04	6.671001e-04	2.695000e-03
Veillonella infantium	3.414000e-04	3.800000e-05	2.157999e-04	3.517000e-04	1.115000e-04	8.919999e-04
Veillonella parvula	0.000000e+00	2.060000e-05	7.825198e-03	0.000000e+00	8.900002e-06	8.259999e-05
Veillonella sp T11011 6	1.047000e-04	1.790000e-05	7.199998e-06	1.050000e-05	2.660001e-05	1.808000e-04
Veillonella tobetsuensis	0.000000e+00	0.000000e+00	0.000000e+00	0.000000e+00	1.465000e-04	0.000000e+00

Supplementary Table S5: Total ARG abundance, RPKM (reads per kilobase per million). Shotgun metagenomic sequencing of *ex-vivo* biofilm community exposed to high ampicillin concentration. Untreated [Control], Ampicillin 10 µg/mL [10.0]

Feature.ID	DonorC-Control-1	DonorC-Control-2	DonorC-Control-3	DonorC- 10.0-1	DonorC-10.0-2	DonorC-10.0-3
patB	135.19171949134	85.2199651679682	57.9484459692142	70.6566432002398	51.9296888196123	51.5585227906257
patA	130.001219637175	83.0483954495842	55.0765749225346	67.1915012386426	49.6536316126694	48.6174862233053
pmrA	71.2225824174355	39.3592025473646	31.4762889711887	43.310468309561	31.7246922669284	22.2867559702414
mefE	0	0	0	0	0	0
CfxA2	0	0	0	0	0	0
mel	173.646227903558	122.643472827005	95.6053789169951	101.670004315314	93.5573221770957	87.3832916611252
ErmB	8.51623657400354	25.1670780915506	10.4243976493402	4.17838984386961	3.08522589445519	9.02327273422123
CfxA3	0	0	0	0	0	0
tetM	141.561605290318	110.987163065547	69.273941663487	66.9542941482476	58.3723667971932	74.5723008455487
aad(6)	0	0	0	0	0	0
CfxA4	0	0	0	0	0	0
CfxA5	0	0	0	0	0	0
lnuC	0	0	0	0	0	0
mgrA	0	0	0	0	0	0
PC1	0	0	0	0	0	0
APH(3')-IIIa	1.2756889240468	1.63086595830738	0	1.27826827518468	2.36210586300846	1.64099445708174
RlmA(II)	106.532114082822	80.3928006025296	59.1435637560895	69.5094670739939	41.7238342891673	50.0682098558671
lsaC	8.16624396059093	8.07753590042562	7.86733235198856	6.38022922053206	3.6359355875831	10.0703691285248
tetB(46)	10.4034044143493	7.5175557034487	5.30749660557496	5.80866172174041	5.06152296520772	5.06965256494813
tetA(46)	11.2240456321272	10.2541963328854	4.30666719078287	3.61884894303837	4.45345650851793	6.05027521567529
tetA(60)	5.08675959682611	3.24666849951216	1.85184899977577	2.71159741872612	2.79620760664129	2.31177451892047
tetB(60)	5.13974667595971	3.24666849951216	3.18929549961382	3.42078443593142	1.37357566642028	1.81193137969442
tetO	4.9940322083423	1.88113835704316	3.40309403865044	2.45738516072055	2.88972112857392	3.85035418185065
SAT-4	3.73544270577239	0	1.48352544180932	1.60414092939514	2.35795349207902	0
TEM-116	0	0	0	0	0	0
CfxA	0	0	0	0	0	0
tetQ	0	0	0	0	0	0

Supplementary Table S6: Relative abundance table of antimicrobial resistance genes (ARGs). Shotgun metagenomic sequencing of *ex-vivo* biofilm exposed to high ampicillin concentration. Untreated [Control], Ampicillin 10µg/mL [10.0]

Gene.ID	DonorC-Control-1	DonorC-Control-2	DonorC-Control-3	DonorC- 10.0-1	DonorC-10.0-2	DonorC-10.0-3
APH(3)-IIIa	0.0015620099197918	0.0027989400201988	0	0.00283586541272528	0.00665424594127732	0.00438399107015834
CfxA	0	0	0	0	0	0
CfxA2	0	0	0	0	0	0
CfxA3	0	0	0	0	0	0
CfxA4	0	0	0	0	0	0
CfxA5	0	0	0	0	0	0
ErmB	0.0104276565839332	0.0431924780225457	0.0256532452827541	0.00926984692426983	0.0086913343756552	0.0241060820892576
lnuC	0	0	0	0	0	0
lsaC	0.00999911015407932	0.0138629041713265	0.0193606012870473	0.014154674510053	0.0102427287469689	0.0269034475662777
mefE	0	0	0	0	0	0
mel	0.212620118750701	0.21048432739864	0.235273856409684	0.225557071505802	0.263558649560612	0.233448424319896
patA	0.159179240981518	0.142530093569668	0.135537124863151	0.149065777576483	0.139878352533898	0.129883818032848
patB	0.165534718487088	0.14625700525145	0.14260446964979	0.156753268872203	0.146290192353865	0.13774093052491
PC1 beta-lactamase (blaZ)	0	0	0	0	0	0
pmrA	0.0872080788287394	0.0675494185231904	0.0774595318339432	0.0960851970375885	0.0893710599772234	0.059540078721588
RlmA(II)	0.13044263051676	0.137972483756922	0.145545517253671	0.154208234185916	0.117539463121101	0.133759491971277
SAT-4	0.00457384120160028	0	0.00365078571655882	0.00355882084154159	0.00664254837181753	0
TEM-116	0	0	0	0	0	0
tetA(46)	0.013743217713267	0.0175985526860076	0.0105982132985243	0.00802849351006325	0.0125457522292067	0.0161635844673664
tetA(60)	0.00622845334788453	0.0055720277618859	0.00455718768756653	0.00601573666677253	0.00787714615540706	0.00617601040846301
tetB(46)	0.0127383883237535	0.0129018497230111	0.0130611395344091	0.0128866398319437	0.014258725307518	0.0135438066092327
tetB(60)	0.00629333307025832	0.0055720277618859	0.00784848990636456	0.00758908317962074	0.00386947530441048	0.00484065680663317
tetM	0.173334288286896	0.19047942649738	0.170475213722215	0.148539528590755	0.164439744604953	0.199223281697997
tetO	0.00611491383373038	0.00322846485588778	0.00837462355432147	0.00545176135426261	0.00814058141608679	0.0102863957140955
tetQ	0	0	0	0	0	0
